# Elucidating dynamic metabolic physiology through network integration of quantitative time-course metabolomics

**DOI:** 10.1038/srep46249

**Published:** 2017-04-07

**Authors:** Aarash Bordbar, James T. Yurkovich, Giuseppe Paglia, Ottar Rolfsson, Ólafur E. Sigurjónsson, Bernhard O. Palsson

**Affiliations:** 1 Sinopia Biosciences, San Diego, CA, USA; 2 Bioengineering Department, University of California, San Diego, La Jolla, CA, USA; 3 Bioinformatics and Systems Biology Program, University of California, San Diego, La Jolla, CA, USA; 4 Center for Systems Biology, University of Iceland, Reykjavik, Iceland; 5 Blood Bank, Landspitali-University Hospital, Reykjavik, Iceland; 6 School of Science and Engineering, Reykjavik University, Reykjavik, Iceland; 7 Department of Pediatrics, University of California San Diego, La Jolla, CA, USA; 8 Novo Nordisk Foundation Center for Biosustainability, The Technical University of Denmark, Hørsholm, Denmark

## Abstract

The increasing availability of metabolomics data necessitates novel methods for deeper data analysis and interpretation. We present a flux balance analysis method that allows for the computation of dynamic intracellular metabolic changes at the cellular scale through integration of time-course absolute quantitative metabolomics. This approach, termed “unsteady-state flux balance analysis” (uFBA), is applied to four cellular systems: three dynamic and one steady-state as a negative control. uFBA and FBA predictions are contrasted, and uFBA is found to be more accurate in predicting dynamic metabolic flux states for red blood cells, platelets, and *Saccharomyces cerevisiae*. Notably, only uFBA predicts that stored red blood cells metabolize TCA intermediates to regenerate important cofactors, such as ATP, NADH, and NADPH. These pathway usage predictions were subsequently validated through ^13^C isotopic labeling and metabolic flux analysis in stored red blood cells. Utilizing time-course metabolomics data, uFBA provides an accurate method to predict metabolic physiology at the cellular scale for dynamic systems.

In recent years, the availability and use of metabolite profiling has substantially increased, allowing for a functional readout of the multitude of influences on cellular biochemistry^1^. Further, time-course metabolomics has allowed the integration of multiple static snapshots to infer biochemical activity, providing more physiological insight, such as in cancer metabolism^2^. Due to the complexities of the associated analytical chemistry, metabolomics has a low signal to noise ratio, and relatively fewer datasets have been generated than for transcriptomics. Thus, formal analysis of metabolomics data is in its infancy as compared with other established omics data types.

One advantage of using metabolomics data is that high-quality, mechanistic models of metabolism are available^3^. Metabolic reaction networks can be represented mathematically and interrogated using ordinary differential equations, metabolic flux analysis, or constraint-based modeling. Constraint-based modeling formalizes biochemical, genetic, and genomic knowledge of cellular metabolism into a mechanistic model and is suited for understanding systems level metabolic physiology without the need for extensive parameterization^4^. Over the past decade, constraint-based modeling methods have developed to better constrain biological systems, in part by including new types of omics data, especially transcriptomics^5^. However, fewer studies have integrated metabolomics data with such networks. To date, dynamic simulations have primarily integrated or modeled extracellular metabolite concentrations^6^,^7^, with some exceptions mentioned in the Discussion^8^,^9^. Without including intracellular concentrations, models can overlook the impact of large intracellular metabolite pools on metabolic flux^10^. Thus, there is a need for methods that can integrate changes in intracellular metabolite data with mechanistic models to accurately predict metabolic physiology under dynamic conditions.

In this study, we present unsteady-state flux balance analysis (uFBA), a constraint-based modeling method and workflow that integrates time-course metabolomics data to predict metabolic flux states for dynamic systems. uFBA and steady-state FBA (henceforth referred to simply as FBA) models were constructed and compared for three dynamic systems: stored human red blood cells (RBCs), stored human platelets, and *Saccharomyces cerevisiae* during anaerobic batch fermentation and carbon starvation. In addition, one classical example of a static system was modeled: *Escherichia coli* during steady-state exponential growth. We find that for the dynamic systems, inclusion of intracellular metabolomics with uFBA provides different and more accurate predictions than FBA. In particular, uFBA predictions for RBC were experimentally validated with isotopic metabolic flux analysis. For the platelet and yeast systems, experimental data from the literature was used to benchmark the modeling results. Finally, the static *E. coli* system served as a negative control, with uFBA and FBA displaying similar predictions.

## Results

### Unsteady-state Flux Balance Analysis (uFBA)

**Figure 1:**
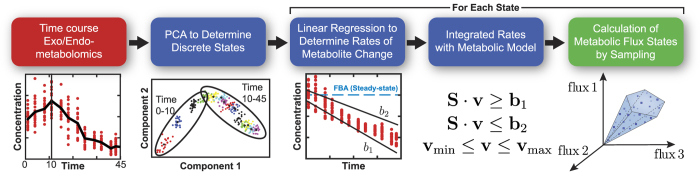
Overview of the uFBA workflow. First, extracellular (exo) and intracellular (endo) metabolite time profiles are split into discrete time intervals of linearized metabolic states using principal component analysis. For each metabolic state, the rate of change of each metabolite is calculated using linear regression, along with the 95% confidence interval (**b**_**1**_ and **b**_**2**_). If the metabolite’s rate of change is significant, the model is updated by changing the steady-state constraint **b** = 0 to a range denoted by **b**_**1**_ and **b**_**2**_. uFBA differs from FBA in that elements of the **b** vector are known and can be used as constraints, but FBA in the absence of such information assumes that these elements are zero (i.e., at steady-state).

The uFBA workflow integrates time-course absolute quantitative metabolomics data with a constraint-based model to predict metabolic flux states. Time-course metabolomics data is often non-linear. The first step of the workflow discretizes non-linear metabolite time profiles into time intervals of linearized metabolic states for piecewise simulation, resulting in a separate model for each metabolic state (Fig. 1). Principal component analysis (PCA) is applied to the time-course metabolomics data to identify the time intervals for which a model will be constructed. For example, the data in Fig. 1 is discretized into two time intervals.

For each time interval, a parameterized model is constructed by (1) determining the rate of change of measured metabolites using linear regression, (2) integrating the calculated rates of change with the constraint-based model, (3) treating the model as a closed system, and (4) reconciling data measurement error and incompleteness through a metabolite node relaxation algorithm to produce a functional model.

The final two steps are different than traditional FBA. uFBA is a data driven approach and aims to make flux predictions solely from time-course metabolomics data. uFBA only allows changes to metabolite levels, including extracellular metabolites for exchange, if the metabolite is measured to be significantly increasing or decreasing in the extracellular (exo) or intracellular (endo) metabolomics data. In principle, if all metabolites are accurately measured over time, the model should simulate. However, due to the experimental infeasibility of data completeness (not all metabolites are measured), additional metabolites will need to accumulate or deplete for proper simulation. To predict which unmeasured metabolites are changing, we developed a relaxation algorithm that makes the model simulation feasible. After all exchange reactions are removed (i.e., a closed system), the metabolite node relaxation algorithm determines the minimum number of metabolites that need to deviate from steady-state for a feasible model. The algorithm and its benchmarking are detailed in the Methods and Supplementary Material.

Once the uFBA model is constructed, most constraint-based modeling analyses can be used, including the maximization or minimization of an objective, flux variability analysis (FVA), and candidate flux sampling. For this study, we focused on Markov chain Monte Carlo (MCMC) sampling^11^ to calculate the probability distribution of flux through every metabolic enzyme in the network. This allowed for the calculation of the most likely flux state of the system.

### Global Differences between uFBA and FBA

To test and validate uFBA, the workflow was applied to four systems: (1) human red blood cells (RBCs) stored up to 45 days at 4 °C; (2) human platelets stored up to 10 days at 22 °C; (3) two strains of *Saccharomyces cerevisiae* during anaerobic batch fermentation and carbon starvation, and (4) *Escherichia coli* during steady-state exponential growth as a negative control. These cases were chosen because they represent diverse systems in terms of physiological dynamics, metabolic network complexity, cell density, data coverage, and timescales (Supplementary Table 1 and Supplementary Fig. 1).

Comprehensive absolute quantitative metabolomics data was obtained from the literature^12^,^13^,^14^,^15^. All four datasets were generated using LC-MS methods, augmented with some extracellular metabolites being measured by HPLC or a blood gas analyzer. Using PCA, the metabolite time profiles for stored RBCs were discretized into three metabolic states, into two states for stored platelets, and into three states for *S. cerevisiae* during mixed glucose/xylose fermentation (Supplementary Fig. 2). The negative control, *E. coli* steady-state growth, was treated as one state. uFBA models were constructed and compared to steady-state FBA models for the corresponding systems and states. The steady-state models were constructed by integrating only the exometabolomics data and allowing for extracellular exchange for unmeasured metabolites with the environment, currently a standard practice.

**Figure 2:**
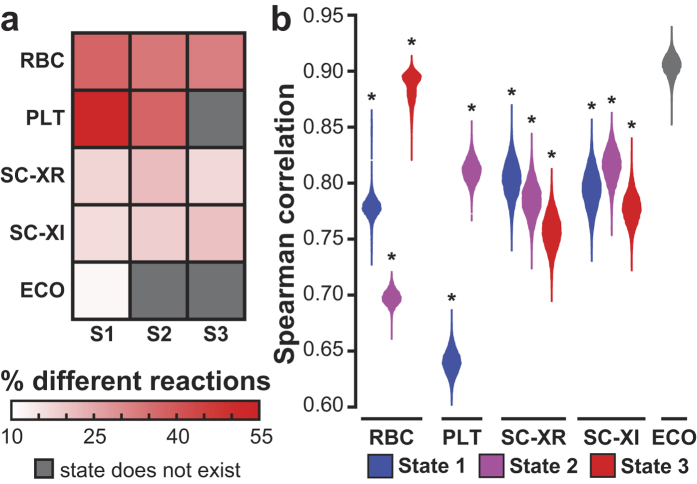
Comparison of uFBA and FBA reaction flux states. (**a**) Percentage of reactions with significantly different fluxes between the uFBA and FBA models. (**b**) The Spearman correlation between uFBA and FBA flux states indicates that differences in flux estimates were not due to uniform increases or decreases in fluxes but a reordering of reactions from high to low flux. The null hypothesis is that the distribution of Spearman correlations is drawn from the same distribution when comparing candidate flux states from uFBA to uFBA or FBA to FBA. *p = 0.0; *E. coli* p = 0.995.

We assessed the global difference between calculated flux states of the uFBA and FBA models (Fig. 2 and Supplementary Fig. 7). We observed considerable differences between uFBA and FBA predictions in the dynamic situations of long-term cell storage or batch fermentation, while fewer differences were observed for *E. coli* during steady-state growth. Further, the detected differences in flux estimates were not due to a uniform increase or decrease of flux across the network as evidenced by the significantly lower correlation of uFBA and FBA calculated fluxes as compared to controls (Fig. 2b). This indicates that the ordering of reactions, from high to low flux, had fundamentally changed in RBCs, platelets, and *S. cerevisiae*.

Reactions with significantly different flux estimates between uFBA and FBA were not uniformly distributed across the subsystems of the various models (see Supplementary Fig. 6). Typically, higher metabolomics coverage of metabolites in a particular subsystem resulted in larger reported flux differences. Further, the number of significantly different reactions within a given subsystem was not constant across metabolic states. This difference is due to the nonlinearity of time-course data, where some metabolite concentrations change during one metabolic state but are at steady-state during another metabolic state.

Outside of MCMC sampling flux states, we assessed whether the size of the solution space had changed by uFBA using flux variability analysis (FVA). We applied FVA to each state of each test case to determine the flux range for each metabolic reaction (flux range = maximum flux − minimum flux). The ratio of the feasible flux ranges of uFBA and FBA are shown (Supplementary Fig. 8). In the RBC and platelet cases, we found that by deviating intracellular metabolites from steady-state, flexibility in the system increased and certain reactions had larger flux ranges for uFBA than for FBA. Overall, however, we found that most of the reactions had equal or lower flux ranges in the uFBA formulation. The lower flux ranges are most likely due to uFBA constraining extracellular metabolite exchanges to only measured data, and not allowing free exchange out of the system.

After a global comparison of uFBA and FBA, we focused on key differences in reaction flux predictions that would have consequences on biological interpretation on metabolic physiology by those investigating the metabolomics data.

### Red Blood Cells

**Figure 3:**
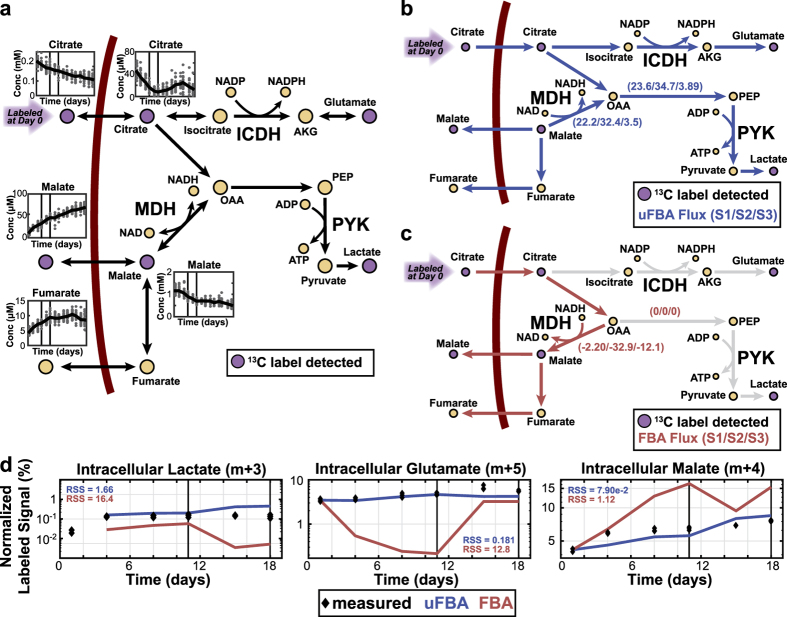
Experimental confirmation of differences in predictions between uFBA and FBA models. (**a**) TCA metabolites and pathways in the RBC metabolic model are shown, including changes in metabolite levels and metabolites found to be isotopically labeled after addition of fully labeled ^13^C citrate. Cofactor producing reactions are shown, while other cofactors and reaction names are omitted. Concentrations are shown as μmol/L of bag or mmol/L of bag. Time spans 0–45 days for insets. (**b**) uFBA (blue arrow) predicts that the depletion of intracellular malate produces extracellular malate and fumarate, while driving lactate production. Extracellular citrate is used to produce glutamate and lactate. Fluxes shown in μM. (**c**) FBA (red arrow) predicts extracellular citrate is used only to produce malate and fumarate and that MDH proceeds in the opposite direction than in uFBA. (**d**) uFBA and FBA predicted fluxes were integrated with a ^13^C MFA “forward” tracer simulation to simulate how labeled citrate would accumulate across the first two metabolic states. The three intracellular metabolites outside of citrate that were observed to be labeled and were absolutely quantified are shown. As fully labeled citrate is used, the only labeled versions of lactate and glutamate are shown. The unlabeled fraction of the metabolite (not shown) is the remainder. For malate, there is a small percentage of m + 3 labeling (see Fig. S11), and the remainder is unlabeled. uFBA predicted more correct labeling patterns than FBA. This is quantitatively corroborated by the residual sum of squares (RSS) for each. Abbreviations: oaa: oxaloacetate; akg: alpha-ketoglutarate; pep: phosphoenolpyruvate; MDH: malate dehydrogenase; PYK: pyruvate kinase; ICDH: isocitrate dehydrogenase. Vertical lines on metabolite time profiles denote the time intervals of the three metabolic states.

There were considerable differences in flux predictions made by uFBA and FBA for RBC metabolism. To hone in on major discrepancies, we focused on metabolic reactions where uFBA and FBA predicted opposite directions of flux. We observed flux reversals in the cytosolic remnants of the TCA cycle reactions. These enzymes had been previously detected in RBC proteomic datasets^16^ (Fig. 3a). Over storage, RBCs uptake citrate (2.32 μM/day during State 1) and secrete malate (1.96 μM/day during State 1) and fumarate (0.300 μM/day during State 1). FBA predicts that nearly all citrate is converted to malate and fumarate (Fig. 3c). This is a reasonable estimation because the flux into and out of the cell is roughly balanced, and the K_eq_ of malate dehydrogenase (K_eq_ = 2.1 × 10^5^) heavily favors metabolite flow in that direction. However, through intracellular metabolite profiling, we discovered that RBCs have a high concentration of intracellular malate (>1 mM). With this additional information, uFBA predicts that the secretion of malate and fumarate are due to the depletion of the large intracellular malate pool (26.1 μM/day, State 1). Further, uFBA predicts the shuttling of the majority of the intracellular malate and citrate into lower glycolysis through oxaloacetate, as well as production of glutamate from citrate (Fig. 3b). The network in Fig. 3a shows all TCA remnant enzymes previously detected in RBCs in proteomic studies^16^ or through literature curation^17^.

We experimentally validated the fate of extracellular citrate by replacing the anticoagulant with fully labeled ^13^C citrate. Measurements of isotope abundance were determined (see Supplementary Data S1) and analyzed using metabolic flux analysis (MFA) tools^18^. As intracellular metabolite levels are changing throughout and the labeling patterns are unstable, traditional “reverse” ^13^C MFA calculations where fluxes are predicted based on isotopic labeling patterns are not applicable. Instead, we completed a “forward” MFA simulation where the isotopic labeling pattern is predicted based on the initial isotopic pattern and the predicted fluxes by uFBA or FBA (see Methods). We compared how well the use of uFBA or FBA fluxes were able to match the isotopic labeling pattern of intracellular metabolites that were detected to be labeled and for which we had absolute quantitation. uFBA produced quantitatively more accurate predictions (lower residual sum of squares (RSS)) than FBA for the isotopic labeling pattern (Fig. 3d and Supplementary Fig. 10). The discrepancy between uFBA and FBA is predominantly due to the depletion of intracellular malate into oxaloacetate, which creates a gradient pushing flux from malate into oxaloacetate.

RBC citrate metabolism can proceed in two directions. First, alpha-ketoglutarate can be formed through aconitase and isocitrate dehydrogenase (IDH). Alpha-ketoglutarate can then form glutamate, which was found to have increasing percentages of isotopic labeling. In this process, NADPH is generated through IDH1, which is the only known isozyme proteomically detected in RBCs^16^. In the second direction, citrate forms oxaloacetate through ATP citrate lyase. An acetyl group is cleaved off during this process forming acetyl-CoA. We detected an increasing labeling percentage of acetylcarnitine (m + 2), which is most probably created from acetyl-CoA. Oxaloacetate can then become aspartate, malate, and lactate, which we found all increasingly labeled. Additional labeled metabolites that were not absolutely quantified and thus not included in MFA, such as acetylcarnitine, are shown in Supplementary Fig. 11.

### Platelets

For the platelet storage data, the major discrepancy between uFBA and FBA models concerned the utilization of the electron transport chain (ETC), particularly in State 1. As this dataset did not have information on oxygen uptake, the metabolite node relaxation algorithm determined the necessary amount based on the rest of the metabolomics data. Based on the measured metabolites, the algorithm was able to accurately predict that oxygen was required for both uFBA and FBA models, but the amount of oxygen needed was only quantitatively predicted on the right order of magnitude for uFBA (see Supplementary Table 7). uFBA predicted that the ETC accounted for 90.2% and 88.8% of ATP generation for States 1 and 2, respectively. However, FBA predicted 0.23% and 64.1% in States 1 and 2, respectively, suggesting that platelets use only glycolysis for ATP generation (and not the ETC) in State 1. Previous experimental studies^19^,^20^ have shown oxygen uptake to be higher in State 1 than in State 2 and that approximately 85% of ATP production during storage is due to the ETC. The uFBA workflow quantitatively predicted the oxygen uptake rate and ATP production. When the FBA models were re-parameterized to not allow free exchange out of the system, the oxygen uptake rate and ETC usage was corrected. The discrepancy between the uFBA and FBA model predictions was determined to be caused by the standard practice in FBA models to allow free exchange of metabolites out of the system, In particular, the reason was free exchange of L-alanine out of the system.

### Saccharomyces cerevisiae

The final dynamic case study used metabolomics data for two *S. cerevisiae* strains engineered to assimilate xylose and fermented in a mixed glucose/xylose culture^14^. One strain consumes xylose through an isomerase (strain XI), while the other consumes xylose through a reductase and a dehydrogenase (strain XR). The PCA identified three metabolic intervals. The first shift differentiated between mixed nutrient metabolism (glucose/xylose, State 1) and xylose as the sole carbon source (States 2 and 3). Using a constraint-based model for yeast metabolism, we found considerable differences between uFBA and FBA flux predictions (Fig. 2). We compared the flux predictions against recently generated ^13^C MFA studies on XI and XR yeast strains^21^,^22^. Although the experimental conditions were not identical (see Supplementary Material), the uFBA model’s predicted flux state (see Supplementary Tables 9–11) resulted in a lower residual normalized error (see Supplementary Table 12) than did FBA when compared with the measured data for the three tested cases: XR growth on glucose (State 1) and xylose (State 3); XI growth on glucose (State 1). XI growth on xylose (State 3) was excluded for comparison as the growth rate of yeast in the ^13^C MFA study was considerably different than in the study used for generating uFBA and FBA predictions.

Further, the uFBA flux predictions provide a systematic method to propose mechanisms for the observed time-course changes in metabolites, rather than use intuition. In particular, the authors postulated that the observed drain of 6-phosphogluconate (6PG) in the XI strain after glucose consumption is due to a reduced flux through the non-oxidative PPP. uFBA instead predicts 6PG decrease to be due to a significant decrease in oxidative PPP activity in State 2. The FBA model did not make a similar prediction. Further, the large decrease in flux through the oxidative PPP predicted by uFBA was confirmed with the ^13^C MFA studies on yeast XI and XR strains noted above. Additional systematically derived hypotheses for observations in the yeast metabolomics data are discussed in the Supplementary Material.

### *Escherichia coli*

The *E. coli* data was used as a negative control because FBA has been traditionally successful in analyzing microbial growth processes based on exometabolomic data alone, in part due to *E. coli*’s balanced growth nature. As expected, uFBA flux predictions deviated less from steady-state flux predictions than did the other test cases (Fig. 2). The similar results demonstrate that the differences observed in the other case studies are not artifacts of the uFBA workflow.

Though flux predictions are not substantially affected, the inclusion of endometabolomics measurements does impact gene essentiality predictions in *E. coli*. Overall results were very similar for the 1,366 genes in the model, but uFBA predicted non-essentiality for 11 genes that FBA predicted to be essential in glucose minimal media. Like other linear programing based constraint-based methods, uFBA cannot explicitly account for metabolites as variables. uFBA incorporates the rate of depleting intracellular metabolites without accounting for the metabolite concentration, thus providing an infinite supply of the metabolite, increasing chances of predicting non-essentiality. This artifact of depleting pools is similar to potential reasons for conflicts in experimental gene essentiality results in minimal media. Experimentally, 17.8% of genes predicted to be essential by uFBA have conflicting experimental results across three studies^23^,^24^,^25^ in glucose minimal media. The 11 differing predictions were enriched in conflicting experimental results (3.06x enrichment, p = 0.0032), suggesting that the measured drains in intracellular metabolites may play a role in conflicting experimental results. The genes were related to NAD and AMP biosynthesis (Supplementary Table 13), which were rescued by the measured depletion of the cofactor pools during growth. The observed discrepancies in gene essentiality calls may be due to differences in plating techniques, the time point for assaying growth, or the chosen growth/no growth threshold. Residual intracellular metabolite pools from LB media before plating may play a role in causing conflicting results. These results suggest that *E. coli* retains higher than required cofactor levels in anticipation of changing environmental conditions, a result consistent with the finding that intracellular concentrations of ATP and NAD pools in *E. coli* are an order of magnitude higher than K_m_’s of their associated enzymes^26^.

## Discussion

Metabolomics data provides a rich detailing of cellular biochemistry. Metabolomics data is becoming readily available, and there is still a need for tools that can integrate such data into mechanistic models to provide a deeper understanding of systems level metabolic physiology. Statistical methods can pinpoint changes or associations but have difficulty elucidating mechanisms. Metabolic modeling techniques can predict which metabolic pathways or enzymes caused the observed statistical changes (i.e., whether upstream or downstream enzymes are more likely the cause of observed behavior), although more detailed, kinetic modeling and metabolic flux analysis are often difficult to construct and parameterize for large cellular networks. Constraint-based models are better suited for studying metabolism at cellular scale, but the steady-state assumption hinders studying dynamic states.

In this study, we present unsteady-state flux balance analysis, a constraint-based modeling method, to study dynamic cellular states. uFBA provides additional utility to existing constraint-based methods that integrate metabolomics data (see Supplementary Material). We identified four test cases and for each, compared uFBA flux predictions with steady-state FBA in order to quantify the advantages of integrating intracellular metabolite concentrations. For the three dynamic systems, we found considerable differences in flux predictions. The size of the metabolic network and data coverage (i.e., what percentage of metabolites are measured) impacts the increased utility of uFBA over traditional FBA as evidenced by the significant differences in RBCs and less significant differences in *S. cerevisiae*. For a more traditional use of FBA (steady-state bacterial growth), we found less difference, confirming that the uFBA is not overly sensitive. Theoretically, in a system where no intracellular metabolites change over time, uFBA and FBA would predict identical flux distributions, indicating that the use of uFBA should be considered if metabolomics measurements are available.

The results presented here have two major implications. First, uFBA quantifies the impact of large and previously unmeasured intracellular metabolite concentration pools on network flux calculations. We experimentally validated a notable uFBA prediction for RBCs using isotopic labeling and metabolic flux analysis. The unexpected complexity of TCA metabolism in human RBCs is biologically notable. RBCs utilize TCA intermediates to produce phosphoenolpyruvate (PEP), most probably through a PEP carboxylase-like mechanism mediated by hemoglobin^27^ which ultimately results to ATP generation by pyruvate kinase. Further, RBCs produce NADH and NADPH outside of glyceraldehyde dehydrogenase and the pentose phosphate pathway through the cytosolic forms of malate dehydrogenase and isocitrate dehydrogenase, respectively. This finding may be of importance in transfusion medicine as some of the FDA approved media additives for RBC cold storage contain large amounts of citrate (20 mM), while other additives do not. The added citrate may affect the red blood cell’s ability to combat oxidative stress during RBC storage^28^. This discovery was only possible through absolute quantitative metabolomics and uFBA. The high levels of intracellular malate (>1 mM) had not been previously observed. Without the intracellular data, the uptake and secretion of metabolites by RBCs was still mass balanced so FBA predictions would have been inaccurate but still feasible. This example shows the importance of combining comprehensive intracellular metabolomics with network calculations. Further, as the labeling percentage of malate is less than 5% at day 1 (Fig. 3d), the concentration of malate is not due to RBC storage in citrate anti-coagulant.

Second, the common practice to allow free metabolic exchange of unmeasured metabolites out of the system can lead to erroneous predictions. For the platelet data, FBA predictions were inaccurate partly due to free exchange of L-alanine out of the system. For the yeast example, differences in FBA and uFBA predictions were partly due to the use of exchange reactions. uFBA aims to be fully data driven and only allows exchange of metabolites out of the system if they were experimentally measured or if the optimization algorithm requires the metabolite to be secreted for model feasibility. With the increasing size of metabolic networks, it requires both intimate knowledge of the cellular system and of constraint-based modeling to identify the modeling inaccuracies caused from metabolic exchanges. The uFBA approach simplifies metabolomics data integration and accurately deals with data inconsistencies through the node relaxation algorithm.

Two other methods for systematically integrating intracellular metabolite concentrations with constraint-based models are available. TREM-Flux^9^ and MetDFBA^8^ have important methodological differences from uFBA. First, TREM-Flux estimates network flux between each two time points, making the approach extremely susceptible to data noise and outliers. The uFBA approach avoids this issue by defining time intervals that represent metabolic states, effectively lowering the chances that noise and outliers affect flux predictions. Second and more importantly, TREM-Flux accounts for data incompleteness by allowing all unmeasured metabolites to deviate from steady state up to the maximum change measured in the metabolomics data. Such an approach provides too much freedom in the optimization problem, making many fluxes inaccurate. uFBA deals with data incompleteness by modifying the fewest number of metabolites using the metabolite node relaxation algorithm.

MetDFBA integrates intracellular measurements with the traditional DFBA approach. In order to deal with the huge complexity of the necessary ordinary differential equations, MetDFBA lumps and removes the majority of metabolic reactions resulting in smaller, core networks. While focusing on specific cellular processes is common in kinetic modeling, it eliminates the ability to interrogate metabolism at the whole-cell level, which is possible with FBA methods. Though uFBA is not as detailed as MetDFBA, it allows for studying metabolism at a comprehensive scale.

The integration of metabolomics data using uFBA has limitations. First, the metabolomics data used to constrain the model must be absolutely quantified using internal standards. Absolutely quantified metabolomics data is often difficult to generate accurately and is more expensive. Second, the increased accuracy of uFBA predictions are determined by how large a percentage of metabolites are measured in the network. In particular, it is important to have metabolite measurements for cofactors and high flux pathways. Third, like other constraint-based methods, uFBA does not explicitly model metabolites as variables and thus total concentrations are not captured. Fourth, organelle specificity of metabolite concentrations is often lacking, requiring modelers to make assumptions on metabolite location. Finally, metabolomics data is noisier than other omics data types. To calculate significant metabolic rates of change, uFBA may require more than three replicates.

uFBA provides a systematic and standardized method to generate hypotheses for the causes of detected changes in metabolite levels over time. uFBA flux predictions are based on unlabeled metabolomics but provide high quantitative accuracy in flux estimates even during dynamic metabolic conditions. These findings are not evident from statistical analysis of the time-course metabolomics alone nor from standard analysis of FBA models. We anticipate that the use of uFBA and the associated workflow will aid in deeper analysis of metabolomics data while also increasing the predictive power of constraint-based models.

## Materials and Methods

All analyses were performed in Matlab (Mathworks, Natick, MA) using the COBRA 2.0 Toolbox^11^. The uFBA method and associated workflow are available as an extension for COBRA 2.0 at (https://opencobra.github.io/cobratoolbox/).

### Data Preparation

Red blood cell (RBC) metabolomics data from normal blood banking conditions using SAGM media was taken from ref. 12. Metabolomics data for platelets retrieved by apheresis during storage under normal blood banking conditions was taken from ref. 13. Metabolomics data for *S. cerevisiae* during mixed culture anaerobic fermentation was taken from ref. 14. Metabolomics data for *E. coli* during exponential growth was taken from ref. 15. Missing values were imputed using a k-nearest neighbor algorithm that takes the weighted average of the five nearest neighbors; this approach has been previously shown to be accurate for metabolomics data^29^. The intracellular and extracellular concentrations were adjusted so that concentrations were in mmol/L of total bag volume for RBCs and platelets, and mmol/gDW for *S. cerevisiae* and *E. coli*. For RBC and platelet datasets, glucose and lactate were measured using a blood gas analyzer. For RBC data, ATP and 2,3 DPG were measured using enzymatic assays. In Bergdahl *et al*. and McCloskey *et al*., changes in key extracellular metabolites in *S. cerevisiae* (glucose, xylose, xylitol, ethanol, and glycerol) and *E. coli* (glucose, acetate, succinate, and formate) were also measured using HPLC. The low-throughput and HPLC measurements were prioritized over mass spectrometry measurements for use in the model. Some metabolite pools were not resolved in the original data and were manually split into individual concentrations based on known physiological ratios (3-Phospho-D-glycerate/D-Glycerate 2-phosphate, pentose sugars, leucine/isoleucine). Due to the rigorous quality control standards within blood banks for those two publications, there was negligible RBC and platelet cell death during storage. Thus, cell death was not included during modeling.

### Principal component analysis and linear regression

Principal component analysis (PCA) was performed on each metabolomics data set in order to objectively determine the time intervals of the discrete, linearized metabolic states. PCA was performed on the standardized Z-scores. Once the time intervals for each state were defined, linear regression was performed in order to estimate the rate of change for each metabolite during that particular state. The 95% confidence interval for each metabolite’s rate of change was calculated for integration with the model. If the 95% confidence interval for a particular metabolite’s rate of change crossed zero, the metabolite was deemed to be at steady-state as there is not enough statistical evidence for the metabolite to be changing. Rates for RBC and platelet were in mmol/L/h, while *S. cerevisiae* and *E. coli* rates were in mmol/gDW/h.

### Constraint-based model integration

The endometabolomics data acquired from the literature was integrated with constraint-based models. The RBC data was integrated with a modified version of the erythrocyte model iAB-RBC-283^17^, which was previously used for building personalized kinetic models^30^. The platelet data was integrated with the platelet metabolic model iAT-PLT-636^31^. The *S. cerevisiae* data was integrated with the *S. cerevisiae* metabolic model iMM904^32^. The *E. coli* data was integrated with the *E. coli* metabolic model iJO1366^23^. The measured growth rate was included as a constraint for *S. cerevisiae* and E*. coli* simulations. Platelet and *S. cerevisiae* metabolic models contain multiple compartments. If metabolites were known to be predominantly from a specific compartment, the metabolomics data was assigned as such. Specifically, tricarboxylic acid cycle metabolites were set to the mitochondria. If no information was available, metabolite concentrations were assumed to be in the cytosol.

### Unsteady-state flux balance analysis (uFBA)

The significant rates of change for measured metabolites for each state (as determined by linear regression) are input to the uFBA method. All four test cases are treated as closed systems, and all exchange reactions were removed from the model. Subsequent steps (see next section) determine other metabolites that can enter or leave the system. The measured metabolomics data is integrated with the model by:


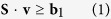



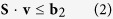


where ***S*** is the stoichiometric matrix, ***v*** is the calculated flux vector, and [**b**_**1**_, **b**_**2**_] represent the 95% confidence interval for each significantly changing metabolite. All unmeasured metabolites are assumed to be at steady-state: **b**_**1**_ = 0 and **b**_**2**_ = 0.

Ideally, all model metabolites would be accurately measured. If so, the model would properly simulate, as all metabolic changes would be accounted for. However, due to experimental limitations, most metabolites cannot be measured, and often those that can be measured are done so unreliably. Optimally, one would measure metabolites that have the highest rate of change relative to the flux going through the associated pathways. However, knowing which metabolites these are may not be possible as such metabolites may change from condition to condition or the system being studied may not be well known. Thus, from any metabolomics dataset, there exists unmeasured intracellular or extracellular metabolites that are not at steady-state that are required to change in order to allow the model to simulate.

To deal with this data incompleteness and data quality issue, we developed an algorithm to reconcile the metabolomics data and the network structure. In brief, the algorithm tries to parsimoniously allow unmeasured metabolites to deviate from steady-state in order to build a computable mode. The approach is detailed in the next section.

Further, a standard practice in FBA is to allow all extracellular metabolites to have free exchange out of the system. As uFBA is a metabolomics driven approach, uFBA only allows exchange of extracellular metabolites out of the system if (1) it is measured to be increasing in the exometabolomics data, or (2) it is required by the metabolite node relaxation algorithm for feasibility.

The remainder of the uFBA modeling formalism follows FBA principles. In particular, a biomass objective function is used in growing cells such as *S. cerevisiae* and *E. coli* to account for the generation of proteins, RNA, DNA, and lipids. Further, metabolite reserves are modeled using sinks. This is used in particular for glycogen stores for *S. cerevisiae* during carbon starvation.

From a mathematical standpoint, the uFBA approach changes the degrees of freedom (DOF) of the system. First, for each “unsteady” metabolite incorporated, the system gains a DOF. Next, all exchange reactions are removed, reducing the DOF by the number of reactions removed. At this point, the metabolic model is most likely infeasible as the system is now over-determined. Previous approaches (i.e. TREM-Flux) deal with this issue by allowing all unmeasured metabolites to deviate from steady-state, increasing the DOF by one for each unmeasured metabolite. The uFBA approach applies a metabolite node relaxation algorithm to parsimoniously deviate unmeasured metabolites from steady-state to minimize the increase of DOF. The system’s increase in DOF is equivalent to the number of metabolites deviated by the algorithm.

### Unmeasured metabolite relaxation from steady-state

Because not every metabolite in the network was measured, we developed an automated method for parsimoniously deviating unmeasured metabolites from steady-state to build a computable model. We term this estimation “metabolite node relaxation”.

For each metabolite that was not measured, two sink reactions are added that allow each of these metabolites to both accumulate (“up” sink reaction) and deplete (“down” sink reaction). Then, an optimization problem determines the minimal number of sinks to retain while still having a computable model. This parsimonious method was chosen under the assumption that cellular systems typically aim to maintain homeostatic levels, which has been previously shown^33^.

To exhaust potential methods, we implemented five different optimization approaches for relaxing the unmeasured metabolite nodes in the system (Equations 3, 4, 5, 6, 7) that all assume parsimony but in slightly different ways. The first technique is an MILP optimization that minimizes the number of unmeasured metabolites relaxed from steady-state (Equation 3):


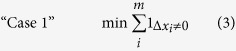


where *m* is the number of unmeasured metabolites in the system and 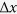 is the deviation from steady-state of the unmeasured metabolites, which is defined as the flux through the sink reaction. Essentially, Case 1 minimizes the increase of the DOF. The second technique is an LP optimization that minimizes the sum of the magnitude of the rate of change of unmeasured metabolites (Equation 4):


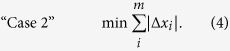


The third technique is also an LP optimization that minimizes both the sum of the magnitude of the rate of change of the unmeasured metabolites as well as the reactions fluxes in the network (Equation 5):


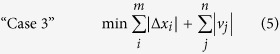


where *n* is the number of intracellular reactions in the system and *v* is the reaction flux. The fourth technique is a QP optimization that minimizes the sum of the square of the rate of change of the unmeasured metabolites (Equation 6):


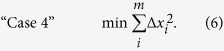


The fifth technique is also a QP optimization that minimizes the sum of the square of the rate of change of the unmeasured metabolites and the square of the intracellular reaction fluxes (Equation 7):


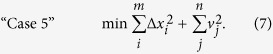


As alternative optima may exist for these optimization problems, especially for Case 1, the given optimization problem is run multiple times for a user-specified number of iterations, not allowing for previous solutions using an integer cut method. For the larger models (platelet, *S. cerevisiae*, and *E. coli*), the termination criterion for optimization for each iteration was set to a relative gap tolerance of 1e-6 or a time limit of 45 seconds. These criteria were chosen based on convergence properties of the solutions as the two parameters were varied.

The multiple solutions are tallied, and a final optimization is run, preferentially weighting unmeasured metabolites that appeared more frequently in the multiple solutions. The sinks associated with the relaxed nodes are retained, while the remaining sinks are removed from the model. 100 iterations were used in this study. A comparison of the accuracy of the optimization approaches in determining necessary relaxations is discussed in the next section. Further, we provide the user with the option to preferentially weight unmeasured extracellular metabolite nodes to be relaxed first, as such an approach is similar to the common practice of modifying extracellular exchanges in FBA models.

Once the final set of unmeasured metabolites to be relaxed from steady-state has been determined, the magnitude of relaxation is addressed. The flux through the retained sink reactions is minimized while allowing the model to simulate. We tested whether increasing this minimum amount affected flux simulations (scaling the minimum bound by 1x, 1.5x, 5x, 10x, or 100x). Sensitivity analysis of this parameter by resampling the reaction fluxes showed that the 1x scaling was too tightly constrained, yielding different results than the higher multiples. However, the higher multiples were very similar to each other. A scaling of 1.5x was used for all analyses in this study as it does not over constrain the network. A full sensitivity analysis for how varying both the cases and the scaling values affects uFBA flux predictions and the comparison of uFBA and FBA is shown in the Supplementary Material.

### Accuracy of unmeasured metabolite relaxation from steady-state

We tested whether the various optimization approaches for parsimoniously relaxing unmeasured metabolites from steady-state was an accurate method for the data incompleteness issue. All five techniques were used to build uFBA models for the RBC metabolomics data. The relaxed nodes for each of the five approaches (see Supplementary Data S2) were compared to a qualitative dataset (Personal Communication with Angelo D’Alessandro) for the same condition that measured many more metabolites than our absolute concentration dataset. The larger dataset had qualitative information on the changes (or lack thereof) for 31 metabolites that were unmeasured in the absolute concentration dataset (see Supplementary Data S3). Based on the time points measured in the qualitative dataset, only states 1 and 3 in the RBC could be compared. Metabolites that increased 2x or decreased 0.5x were deemed to be changing in the qualitative dataset.

The criterion for selecting the best optimization technique was minimizing the number of cases where a metabolite was incorrectly relaxed from steady-state. This criterion was chosen as it is more detrimental to over relax than to under relax, because it allows the model to have many new feasible flux states that may be erroneous. We found the MILP formulation (Case 1) to be the best method for this criterion. Further, the MILP formulation was also found to have the overall best accuracy (see Supplementary Table 3). For all analyses in the main text, the MILP formulation is used.

### Construction of FBA models

The FBA models constructed for control comparison against the uFBA models followed the same workflow as the uFBA model, except that only the exometabolomics data was integrated. Further, in order to satisfy the intracellular steady-state requirements for FBA, models are typically allowed to have free extracellular metabolite exchange out of the system. This practice was used for the FBA models in this study. Still, the optimization algorithms for node relaxation were required and were used in the same manner as that for uFBA. A list of relaxed nodes for the FBA models is provided (see Supplementary Data S2).

### Markov chain Monte Carlo (MCMC) sampling and gene essentiality

The FBA and uFBA models were sampled using Markov chain Monte Carlo (MCMC) sampling methods^11^. We sampled 5,000 points for the RBC model and 10,000 points for the platelet, *S. cerevisiae,* and *E. coli* models; all models were sampled until the mixed fraction was below a threshold of 0.54. The sampling distributions for each reaction were deemed significantly different between modeling formulations (uFBA vs FBA) if the two distributions overlapped by less than 5%. The correlations which are represented as histograms in Fig. 2b of the main text were calculated using the Spearman correlation of two sampled flux vectors. The process was repeated 5,000 times for the RBC model and 10,000 times for the platelet, *S. cerevisiae*, and *E. coli* models to account for all generated sample points. For all determining significant differences and Spearman correlations (including controls), only reactions that were in both models and were not involved in type 3 loops were compared.

Gene essentiality predictions were completed with COBRA 2.0^11^. The threshold for growth was set to 1% of the growth rate experimentally determined in the publication of the metabolomics dataset^15^. All bounds and constraints that conflict with the zero vector being a feasible solution were modified to allow for proper simulation. Metabolic exchange out of the system for the uFBA model was also allowed. Computational predictions were compared to four data sets of glucose minimal media in three publications^23^,^24^,^25^.

### ^13^C labeling citrate experiment

A healthy donor was recruited and, after obtaining informed consent, red cells were obtained using ISO 9001:2008 certified red cell isolation protocols at the Landspitali-University Hospital Iceland Blood Bank. The study and all associated methods were approved by The National Bioethics Committee of Iceland and the Icelandic Data Protection Authority. The methods were carried out in accordance with the guidelines and regulations outlined by those committees.

Isolated red cells were added to 65 ml of modified Citrate Phosphate Dextrose (CPD) solution containing 105 mM uniformly labeled ^13^C citrate (~78% m + 6, 1.5% m + 5, 20.5% m + 0) in place of unlabeled citrate in regular CPD SAGM media. Sodium hydroxide was use to match ionic strength imposed by trisodium citrate in regular CPD SAGM (Fenwal, Lake Zurich, IL, USA). As a negative control, red cells were stored in unmodified CPD SAGM. RBC units were stored at 4 °C. 5 mL samples were removed at ten time points and aliquoted accordingly for subsequent quality control assessment and metabolomics analysis. The experiment was run for 31 days, as the shift in metabolic dynamics from State 2 to State 3 occurs much earlier (day 17).

Red cell blood banking quality control assessment was performed immediately by ABL90 FLEX blood gas analyzer (Radiometer, Copenhagen, Denmark) determining pH, pO2, pCO2, total hemoglobin, [K+], [Na+], [Ca2+], [Cl−], [Glucose], and [Lactate]. A XN-1000 hematology analyzer (Sysmex, Norderstedt, Germany) was used to record RBC count, white blood cell count, platelet count, hemoglobin, % hematocrit, total mean cell hemoglobin, mean cell hemoglobin concentration, and red cell distribution width. Labeled samples were found to have similar QC properties as control units.

Metabolomic analysis was performed using a previously reported method^13^,^34^ based on ultra high performance liquid chromatography (UHPLC) (Acquity, Waters, Manchester, UK) coupled with a quadrupole-time of flight mass spectrometer (Synapt G2, Waters). Chromatographic separation was achieved by hydrophilic interaction liquid chromatography (HILIC) using an Acquity amide column, 1.7 μm (2.1 × 150 mm) (Waters).

500 μL of RBC sample were used for metabolomics analysis and supernatant and cells were separated by centrifugation (1600 g, 4 °C, 15 min). Immediately after centrifugation, cell-free supernatant was removed from centrifuged tubes and collected in separates tubes and processed as previously described^13^,^34^.

Data integration of targeted compounds was achieved by using TargetLynx (Waters). Raw data was then corrected for natural abundance of ^13^C isotopes using IsoCor (MetaSys, Toulouse, France)^35^ that afforded the % isotopes that exceed natural abundance and the corrected isotopic distribution.

### Metabolic Flux Analysis

Metabolic flux analysis (MFA) was completed using the INCA software suite^18^ in order to compare the measured isotopic labeling patterns to the flux predictions of the uFBA and FBA models. An isotopic model was constructed for the MFA simulations in which all reactions were split into irreversible reactions and metabolic reactions not directly related to citrate metabolism or production of the labeled metabolites were omitted (see Supplementary Data S4). For uFBA associated MFA simulations, accumulation or depletion of metabolites was modeled using sinks. If a labeled intracellular metabolite was decreasing, the associated sink was labeled at the percentage of the initial labeling pattern for that particular state.

The INCA software suite allows for two basic types of simulations: a “forward” simulation in which the labeling pattern is predicted for a given flux state, and a “reverse” simulation in which the flux state of the network is calculated based on fitting the experimentally measured labeling pattern. The traditional “reverse” MFA simulation cannot be used when both metabolite levels are changing and the labeling pattern is unsteady. Instead, the “forward” simulation was used, which takes as input: (1) measured intracellular metabolite concentrations, (2) the labeling pattern of the metabolites, and (3) the flux state of the network. The mean flux state of the network as calculated by MCMC sampling for each of the metabolic states for each of the uFBA/FBA models was used as the initial flux state labeling pattern on Day 1.

The “forward” MFA simulations were completed for the first two states, where the labeling pattern approaches steady-state. First, the initial labeling pattern (Day 1) and the estimated uFBA fluxes were inputted and the labeling pattern was simulated for the duration of State 1. Next, the final predicted labeling pattern and the uFBA fluxes for State 2 were simulated for the duration of State 2. The labeling pattern reached steady-state by State 3 and is not included as there were little differences between uFBA and FBA. The same workflow was also applied to the FBA fluxes for comparison.

To assess goodness of fit between the measured data and the simulated data, the residual sum of squares (RSS) was calculated for each of the four metabolites for which we had absolute concentrations and labeling data (lactate, glutamate, malate, and citrate). The RSS was calculated by


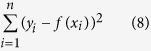


where *y* is the experimental value, *f(x*) is the tracer simulation predicted value and the range of *i* contains all measured time points and each replicate is treated as a separate value. We evaluated the relative difference in RSS between the uFBA and FBA simulations to demonstrate the difference in accuracy of predictions. All RSS calculations were done with log10 transformed abundances.

### Comparison to experimental flux states

Candidate flux states for the uFBA and FBA models were determined using MCMC sampling. The mean sampling vector was compared to measured flux values for the XR strain growth on glucose and xylose^21^ and for the XI strain growth on glucose^22^. Though the experimental conditions were not identical, comparisons were made when the yeast growth rate were similar across the respective experimental conditions. Fluxes from metabolic reactions involved in type III pathways that are known to result in erroneous flux predictions were omitted for the comparison to measured data. In order to evaluate the error of the predicted flux states, we calculated the normalized Euclidean distance given by:


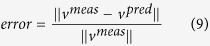


where *v*^*meas*^ is the flux vector from MFA measurements and *v*^*pred*^ is the flux vector from the uFBA and FBA models.

## Additional Information

**How to cite this article:** Bordbar, A. *et al*. Elucidating dynamic metabolic physiology through network integration of quantitative time-course metabolomics. *Sci. Rep.* **7**, 46249; doi: 10.1038/srep46249 (2017).

**Publisher's note:** Springer Nature remains neutral with regard to jurisdictional claims in published maps and institutional affiliations.

## Supplementary Material

Supplementary Text

Supplementary Dataset S1

Supplementary Dataset S2

Supplementary Dataset S3

Supplementary Dataset S4
