# Supplementary material for: Elucidating dynamic metabolic physiology through network integration of quantitative time-course metabolomics
Source: Sci Rep. 2017 Apr 7;7:46249. doi: 10.1038/srep46249 (PMC5384226; doi:10.1038/srep46249)
Supplement: Supplementary Text [file srep46249-s1.pdf]

Supplementary Material for “Elucidating dynamic metabolic physiology through network integration of quantitative time-course metabolomics”

A Bordbar*, JT Yurkovich*, G Paglia, O Rolfsson, Ó Sigurjónsson, and BO Palsson

Table of Contents

1. Data characterization ... 3
2. Discretizing time-course metabolomics with principal component analysis ... 3
3. Model construction parameters ... 4
4. Sensitivity analysis of varying metabolite node relaxation parameters ... 5
5. Accuracy of metabolite node relaxation ... 5
6. Observed differences between FBA and uFBA by subsystem ... 6
8. RBC ^{13}C MFA citrate labeling experimental validation ... 6
9. uFBA differences in the RBC Rapoport-Luebering shunt ... 7
10. Generating hypotheses for observed changes in the S. cerevisiae metabolome ... 8
11. Gene essentiality of Escherichia coli ... 9
12. Documentation for uFBA COBRA method ... 9

Supplementary Figures ... 12
- Supplementary Figure 1 ... 12
- Supplementary Figure 2 ... 13
- Supplementary Figure 3 ... 15
- Supplementary Figure 4 ... 16
- Supplementary Figure 5 ... 17
- Supplementary Figure 6 ... 18
- Supplementary Figure 7 ... 19
- Supplementary Figure 8 ... 20
- Supplementary Figure 9 ... 21
- Supplementary Figure 10 ... 22
- Supplementary Figure 11 ... 23
- Supplementary Figure 12 ... 24

Supplementary Tables ... 25
- Supplementary Table 1 ... 25
- Supplementary Table 2 ... 26
- Supplementary Table 3 ... 27
- Supplementary Table 4 ... 28
- Supplementary Table 5 ... 29
- Supplementary Table 6 ... 30
- Supplementary Table 7 ... 31
- Supplementary Table 8 ... 32
- Supplementary Table 9 ... 33
- Supplementary Table 10 ... 34
- Supplementary Table 11 ... 35
- Supplementary Table 12 ... 36
- Supplementary Table 13 ... 37

References ... 38

1. Data characterization

We chose four test cases that represent diverse systems in order to best characterize the difference between uFBA and steady-state models (**Supplementary Table 1**). Differences in datasets include the amount of carbon, nitrogen, oxygen, sulfur, and phosphorus inside and outside of the cells, based on the measured small metabolites (**Supplementary Fig. 1**). In RBCs, 29.1% of carbon in the system was located in the cell. In platelets, roughly 2.87% of measured carbon was in the cells. In *S. cerevisiae*, only 0.11% of measured carbon was in the cells, while 0.963% of measured carbon was intracellular in *E. coli*. The low levels of intracellular carbon in the three latter cases is due to the low volume ratio of cells to the total volume. However, over time in the *S. cerevisiae* experiments all the glucose was consumed by the cells changing the extracellular/intracellular ratio. This change is reflected in the drop in Spearman correlation of uFBA to FBA fluxes in the later states for *S. cerevisiae* (**Fig. 2b**). Nitrogen, sulfur, and phosphorus were predominantly located in the cells across all three cases. Each test case varied in average cell size and the volume fraction in the system that was cells (**Supplementary Table 1**). The complexity of cellular metabolism varied between test cases, which affected the percentage of the total metabolome that we were able to measure. Further, the systems varied in how much the measured metabolites deviate from steady-state. In RBCs, platelets, and *S. cerevisiae*, >80% of the measured metabolites that were in the model were not at steady-state in at least one of the metabolic states. In *E. coli*, roughly 45% of measured metabolites were not at steady-state. This is to be expected as the *E. coli* measurements were made during a particular state of exponential growth. The timescale of the measurements also varied, where RBC and platelet measurements were made over days while *S. cerevisiae* and *E. coli* measurements were made over hours. Thus, the three test cases chosen were quite diverse.

The differences in uFBA from steady-state modeling in the main text, or lack thereof, are reflections of the different experimental conditions mentioned above. In particular, *E. coli* uFBA results were very similar to FBA modeling in part due to: (1) the chosen experimental condition that was not dynamic, (2) the percentage of measured metabolites that deviate from steady-state, and (3) the much smaller volume of *E. coli*. We anticipate that if *E. coli* metabolomics data is procured in a more dynamic situation and a higher percentage of intracellular metabolites were measured, the differences between uFBA and FBA would be comparable to those seen in the other test cases. Further, we anticipate that using uFBA on mammalian cell culture metabolomics data will be of interest as those cell types are 10-100x larger than RBCs.

2. Discretizing time-course metabolomics with principal component analysis

Constraint-based modeling does not explicitly model metabolite concentrations. Thus, dynamic metabolite profiles cannot be directly integrated with such models, as would be the case with kinetic models. Still, the rates of metabolite change can be integrated. As constraint-based models are linear models, integrated rates of metabolite changes should be close to linear, as

other behaviors (e.g., oscillations) cannot be accurately modeled. Thus, we used principal component analysis (PCA) to objectively discretize non-linear time-course data into time intervals that were predominantly linear in terms of metabolite change for piecewise analysis. PCA was performed on the standardized Z-scores of the RBC, platelet, and yeast raw data and determined that there were three metabolic states in the RBC data (days 1-11, days 11-18, and days 18-43), two metabolic states in the platelet data (days 0-3 and days 3-10), and three metabolic states in the yeast data (hours 14.5-18.5, hours 18.5-22, and hours 22-38.5) (**Supplementary Fig. 2**). The *E. coli* data was chosen and modeled as one metabolic state as there were only 3 time points, all during exponential growth. Further, manual inspection of RBC, platelet, and yeast metabolites confirmed that PCA was able to separate out the majority of nonlinear behaviors of metabolite level change into predominantly linear states; examples are shown in **Supplementary Fig. 2**.

3. Model construction parameters

The extracellular metabolites were preferentially weighted to deviate from steady-state first (eWeight = 1e6, see documentation of MATLAB code). 100 iterations of the node relaxation algorithm were used for all models. Some metabolites were hardcoded not to be allowed to deviate from steady-state. This was done for metabolites that were known to have very low concentrations or not be present in the conditions in which the data was procured (**Supplementary Table 2**).

Additional adjustments, RBC: NADP and NADPH measurements were ignored as they were unreliable assays. The minimal change in cysgsh[e] in state 2 and 3 was not ignored, even though it was not significant.

Additional adjustments, PLT: The reaction for glutathione dehydrogenase (GTHDH) was added so that the model could reduce dehydroascorbate to ascorbate. The transport reactions for L-histidine and L-lysine were both changed to be reversible. Glutathione peridoxase (GTHPi), extracellular glutathione reductase (GTHPe), and mitochondrial glutathione reductase (GTHPm) were made irreversible. For the PLT FBA model, a sink was created for ncam[e] in both states.

Additional adjustments, SC: Necessary reactions for xylose assimilation were added to the model for each strain as specified in Bergdahl et al.[1] Oxygen was not allowed into the system in order to simulate anaerobic conditions. Ions, protons, and water were allowed to exchange freely. The intracellular measurements for 1,3-bisphosphate glycerate were ignored similar to the original publication. Extracellular measurements were reported as mean and standard deviation for three replicates. Three pseudo-replicates were generated on a normal distribution around the mean and standard deviation to calculate metabolite rates of change. The process was repeated 1000 times and averaged for the final calculated 95% confidence interval.

Additional adjustments, ECO: The measurements for inositol (both intracellular and extracellular) were ignored due to measurement error. Ions, protons, and water were allowed to

exchange freely. Exchange of CO_2 and methanol out of the system was freely allowed.

4. Sensitivity analysis of varying metabolite node relaxation parameters

Using the RBC data, we completed a sensitivity analysis on the two parameters necessary for metabolite node relaxation: (1) the optimization approach ("*Case*"), and (2) the scaling factor of the minimum flux bounds for a feasible solution ("*lambda*"). See Methods for further explanation of parameters. First, we determined the number of metabolites that were deviated from steady-state by each *Case*, as well as the necessary magnitude of deviation from steady-state to produce a feasible model (**Supplementary Fig. 3**). Cases 1 and 2 relaxed the fewest metabolites while Cases 1, 2, and 4 had the smallest total magnitude of relaxation.

Next, we compared how varying both parameters affected flux predictions for the three RBC metabolic states. Flux states were computed by MCMC sampling for the five *Cases* and for five different *lambda* values (1x, 1.5x, 5x, 10x, and 100x of minimum magnitude necessary for a feasible model). 25 models were built for each pairing of the two parameters. The percentage of significantly different reactions was determined for the 25 models through a pairwise comparison (**Supplementary Fig. 4**). We found that varying the optimization approach can yield changes in results. However, the biggest changes occur when the *lambda* value is set to a stringent value of 1. The higher *lambda* values had much less change between each other and also lowered the difference between Cases. These results indicate that a particular optimization approach has to be well chosen. The accuracy of the various optimization approaches is compared to an independent dataset for validation in the next section.

Further, we compared uFBA and FBA to all 25 combinations of *Cases* and *lambdas* (**Supplementary Fig. 5**). The difference between uFBA and FBA was relatively constant across all 25 parameter combinations.

5. Accuracy of metabolite node relaxation

We assessed which of the five metabolite node relaxation optimization approaches is most accurate for determining unmeasured metabolites that should deviate from steady-state. A qualitative dataset was procured (personal communications with Angelo D'Alessandro) and used for validation. This dataset measured 31 metabolites that were in the RBC model (see **Supplementary Data S3**) but were unmeasured in the absolute quantitative dataset used to build the uFBA models in the main text. The qualitative dataset had seven measurements from days 1 to 43. The time points did not line up with the metabolic states determined by PCA (see **Supplementary Fig. 2**), as our absolute quantitative dataset measured metabolites every 3-4 days. Thus, we only compared State 1 with days 1-15 and State 3 with days 22-43.

We believe that the best optimization technique would minimize the number of incorrectly relaxed metabolites from steady-state, as erroneous relaxations would lead to additional degrees

of freedom of the network. As we had three categories (accumulate, deplete, steady-state), we determined the binary classifier accuracy of the methods three times, where the positive prediction was each of the categories. We primarily focused on the category where steady-state is the positive prediction and determined the optimization approach with the highest true positive rate (TPR = TP / (TP + FN)). Case 1 had the highest TPR in both metabolic states. Further, we looked at the average of the overall accuracy of all three categories which was also Case 1 (**Supplementary Table 3**). Therefore, we selected Case 1 for this study. The final code contains all optimization approaches.

6. Observed differences between FBA and uFBA by subsystem

The underlying Spearman correlation distributions used to generate the violin plots in **Fig. 2b** are shown in **Supplementary Fig. 7**. Determination of significantly different Spearman correlations between uFBA and FBA was determined by comparing the distribution against the combination of control distributions for the correlations of uFBA vs uFBA and FBA vs FBA.

7. Additional observed differences between FBA and uFBA using other constraint-based methods

In the main text, MCMC sampling is used to calculate differences between uFBA and FBA formulations. Here, we further characterize the difference of modeling formulations using linear optimization for various user-defined objective functions and assessing the solution space, using flux variability analysis (FVA).[2] First, we chose several objective functions for the four test cases and calculated the maximum flux through those reactions (**Supplementary Table 4**). Similar to MCMC sampling results, there were considerable differences for flux values in the dynamic test cases (RBC, platelet, and yeast). There was little difference in *E. coli* results. Second, we assess the change in the solution space by using the uFBA formulation. We applied FVA to each State of each test case to determine the flux range for each metabolic reaction (flux range = maximum flux – minimum flux). FVA was applied without first optimizing an objective function. For illustrative purposes, the ratio of the feasible flux ranges of uFBA and FBA are shown (**Supplementary Fig. 8**). In the RBC and platelet case, we found that certain reactions had larger flux ranges using uFBA. For the other cases, most of the reaction had equal or lower flux ranges in the uFBA formulation.

8. RBC ^{13}C MFA citrate labeling experimental validation

In order to determine whether uFBA or FBA provided more accurate flux predictions for RBC utilization of TCA intermediates, we conducted an isotopic metabolic flux analysis (MFA). For blood banking, whole blood is initially placed in citrate-phosphate-dextrose (CPD) solution. Next, the whole blood is separated into its individual blood component, such as RBCs in satellite

bags. During cell separation, RBCs are placed in saline-adenine-glucose-mannitol (SAGM) solution, which does not contain citrate. We replaced the anticoagulant citrate in the initial CPD bag with ^{13}C fully labeled citrate (i.e., all 6 carbons labeled, 80% m+6). Metabolomics measurements of isotopically labeled metabolites began nearly 24 hours after the donation, processing, and placement in SAGM, meaning some of the label had already propagated into intracellular metabolites. We were able to isotopically track 96 metabolites, which had a high overlap with the metabolites measured in the original study.[3] Of these tracked metabolites, we saw reliable signals of ^{13}C labeling in eight metabolites (**Supplementary Table 5**). Reliable signals were those for which (1) the labeled signal was a high percentage of total signal for that metabolite or (2) the labeled signal had a clear increase or decrease.

uFBA and FBA provided very different predictions for how RBC citrate metabolism occurs during cold storage, as uFBA takes into account the intracellular metabolic changes (**Fig. 3a**). To quantitatively assess which method provided more accurate flux estimates, we completed ^{13}C MFA. As intracellular metabolite levels are changing throughout the labeling experiment and the labeling patterns are unstable, traditional “reverse” ^{13}C MFA calculations are not applicable. Instead, we completed a “forward” MFA simulation (see **Methods**). We found that uFBA fluxes were quantitatively more accurate in predicting the isotopic labeling pattern for all intracellular metabolites that we found to be labeled and that we had absolute quantitation (**Fig. 3b** and **Supplementary Fig. 10**). Labeled metabolites that were not absolute quantified (and thus not included in MFA) are shown in **Supplementary Fig. 11**.

9. uFBA differences in the RBC Rapoport-Luebering shunt

Other intracellular pools were also observed to significantly affect the metabolic flux state of the RBC. The most significant of these pools, 2-3-diphosphoglycerate (2,3-DPG), is located in the Rapoport-Luebering shunt in glycolysis, through which 1,3-bisphosphoglycerate (1,3-BPG) is converted to 2,3-DPG through the diphosphoglyceromutase (DPGM) reaction. This pathway is important for regulating oxygen binding to hemoglobin. The FBA model predicts that DPGM occurs in the forward direction (1,3-BGP to 2,3-DPG) and that 2,3-DPG proceeds to 3-phosphoglycerate. However, the uFBA model predicts that DPGM occurs in the reverse direction, which is surprising as the reaction is thought to be irreversible and has a large K_{eq}. This prediction is due to (1) the measured depletion of 2,3-DPG, but more importantly to (2) the measured buildup of ATP in State 1. Based on the model, the only way the RBC can produce the measured ATP buildup rate would be to run DPGM in reverse (see **Supplementary Fig. 12**). This reaction may be driven by the need to buffer protons generated by glycolysis. The RBC bag becomes acidic over time (pH < 6.3 by day 42) and the estimated production rate of H^+ in state 1 by glycolysis is roughly the same rate as the 2,3-DPG depletion rate in State 1. The DPGM reaction in the Rapoport-Luebering shunt may be driven by the need to buffer protons generated by glycolysis. The RBC bag becomes acidic over time (pH < 6.3 by day 42) and the estimated production rate of H^+ in state 1 by glycolysis is roughly the same rate as the 2,3-DPG depletion rate in State 1. The uFBA predicted solution discussed in the main text is also consistent with the latest, approved media formulation for RBCs (AS-7). AS-7 is an alkaline solution which has

been shown to maintain 2,3-DPG levels for longer than traditional media formulations,[4] such as the SAGM media used in the metabolomics data study.

An additional mechanism for the depletion of 2,3-DPG in RBCs during storage has been proposed.[5] This mechanism involves the activity of another enzyme, MIPP1, which cleaves the other phosphate off 2,3-DPG and produces 2-PG. This proposed mechanism for depletion of 2,3-DPG was computationally tested with uFBA by adding the reaction into the RBC model. MCMC sampling predicted that the flux through the proposed MIPP1 reaction is 20-fold lower than the flux through DPGM (see **Supplementary Table 6**). This reaction was also not predominantly used because ATP cannot be generated from this mechanism.

10. Generating hypotheses for observed changes in the *S. cerevisiae* metabolome

Metabolomics data for two isogenic strains of *S. cerevisiae* that are able to consume xylose was procured from Bergdahl et al.[1] The two strains used different means of converting xylose to xylulose. The XI strain used xylose isomerase to directly convert xylose to xylulose. The XR strain used xylose reductase to convert xylose to xylitol and then a xylitol dehydrogenase to convert xylitol to xylulose. In the process, the XR strain utilizes NADPH for the reductase and generates NADH for the dehydrogenase. The two strains were grown in the same mixed glucose/xylose conditions, but had slightly different metabolic pathway usages.

There were many reactions that were significantly different between uFBA and FBA (**Fig. 2a**), which led to significantly different flux vectors as indicated by Spearman correlations (**Fig. 2b**). A very large difference between FBA and uFBA was the use of glucose. In State 1, where large amounts of extracellular glucose were available, uFBA utilized much more glucose for oxidative pentose phosphate pathway (PPP) as compared to FBA (**Supplementary Table 8**). The uFBA flux split is much more in line with ^{13}C metabolic flux analysis splits for PPP in yeast during glucose fermentative growth.[6]

We used the uFBA model to derive hypotheses for several of the observations that Bergdahl and colleagues made for their metabolomics data. Model hypotheses are systematic and standardized. They often agreed with assessments by the authors, but sometimes did not, as discussed in the main text. Particularly, Bergdahl et al. postulated that the decrease in 6-phosphogluconate is due to a reduced flux through the non-oxidative PPP. uFBA instead predicts this is due to a significant decrease in oxidative PPP activity in State 2 after external glucose depletion for the XI strain (**Supplementary Table 8**). uFBA does not predict as much a drop in oxidative PPP in the XR strain in State 2, allowing maintenance of a lower level of 6PG. These results are consistent with recent ^{13}C MFA studies on yeast XI and XR strains measuring oxidative PPP flux grown on glucose and xylose[7,8]. These ^{13}C labeled experiments represented similar but not identical experimental conditions. Metabolomics data used for uFBA came from mixed glucose/xylose cultures, where cells predominantly used glucose in state 1 and only used xylose

in state 3. Labeling experiments were done in glucose only or xylose only cultures. Thus, the flux predictions would not be expected to perfectly match experimental data because the models were informed with different data. In particular, the XR strain maintains oxidative PPP, even when glucose is depleted. FBA is unable to recapitulate these results.

Second, the authors postulated the accumulation of PEP in State 3 for the XI strain to be due to decrease pyruvate kinase (PYK) activity. uFBA flux predictions were consistent with this assertion as PYK flux significantly dropped in State 3 of the XI strain but not the XR strain. Third, the metabolomics data indicated that in State 3 the XI strain has a drop in acetyl-CoA levels, while the XR strain maintains acetyl-CoA levels. According to uFBA, this is due to a significant decrease in mitochondrial pyruvate dehydrogenase for the XI strain, while XR maintains pyruvate dehydrogenase levels to generate acetyl-CoA.

11. Gene essentiality of *Escherichia coli*

Outside of looking at flux states using MCMC sampling, FVA, and optimization of objective functions, we tested the differences in gene essentiality between uFBA and FBA. As uFBA adds artificial sinks for intracellular metabolites that are decreasing, erroneous gene essentiality predictions are possible. However, as discussed in the main text, we found that gene essentiality between the two models was very similar except for 11 metabolic genes, which are shown in **Supplementary Table 9**. The 11 genes were enriched with a set of genes that show conflicting experimental results across multiple studies.[9-11] Four of the genes were essential in LB medium,[10] showing that uFBA made erroneous predictions. Of the remaining seven genes, six had conflicting experimental results in the three studies.

12. Documentation for uFBA COBRA method

uFBA was implemented in Matlab R2014b and integrated with the COBRA Toolbox 2.0.[12] The method is freely available online at: (http://opencobra.github.io/cobratoolbox). Here, we give a more in-depth description of the inputs and outputs of the function to build the uFBA model based on measured rates of change in a particular metabolic state. The primary function for uFBA is called “buildUFBAmodel.m”.

Required inputs	Description
model	A COBRA model structure containing (at minimum) the following fields: S, b, lb, ub, mets, rxns
metNames	A cell array containing the model IDs of the measured metabolites that will have bounds set by the algorithm. These metabolites should correspond to model.mets. Note: measured metabolites that were not significantly changed over time should also be included.
changeSlopes	A vector (length(metNames) x 1) that contains the mean rate of change (the slope from linear regression) for each metabolite in metsMeasured.

changeIntervals	A vector (length(metNames) x 1) that contains the difference between the mean slope of change and the upper bound of the 95% confidence interval for each slope in changeSlopes.
ignoreSlopes	A binary vector (length(metNames) x 1) that instructs which changeSlopes to be ignored (ignore if 1). Metabolites were ignored if the values of the slopes were not significant based on linear regression (i.e., if slope value +/- the interval crossed zero).
objRxn	The objective reaction (corresponding to model.rxns) for the new uFBA model.
**Optional inputs**	**Description**
metNoSink	A cell array of metabolites (corresponding to model.mets) that should not have a sink added, typically for metabolites where the concentration is known to be zero. Default is an empty cell array.
metNoSinkUp	A cell array of metabolites that should not have a sink added in the up direction (which would allow metabolite accumulation). Default is an empty cell array.
metNoSinkDown	A cell array of metabolites that should not have a sink added in the down direction (which would allow metabolite depletion). Default is an empty cell array.
conflictingMets	A cell array of intracellular metabolites (corresponding to model.mets) where the intracellular rates conflict with extracellular rates and the model cannot compensate through biosynthesis of the metabolite or use of the flux in other pathways. Typically, only necessary for very simple cell types (e.g. RBCs). The intracellular rate is adjusted to the extracellular to allow the model to simulate. Default is an empty cell array.
solvingStrategy	One of ['case1','case2','case3','case4','case5'] which correspond to the 5 node relaxation techniques discussed in the main text. Default value is the first LP technique, 'case2'.
lambda	A multiplicative relaxation away from the minimum allowed deviation from the steady state model. Default value is 1.5.
numIterations	The number of iterations for the integer cut optimization method. Default value is 100.
timeLimit	The time limit for the solver during the numIterations optimization loop. Default value is 30 seconds.
eWeight	A weighting factor for preferential selectin of extracellular sinks over intracellular during node relaxation. Default value is 1e4. If no weighting is preferred, eWeight should be set to a value of 1.
**Outputs**	**Description**
model	The final uFBA model.
metMeasurements Applied	Metabolites that had measurements applied.
relaxedNodes	A cell array which contains three columns: (1) which metabolites were relaxed from steady-state; (2) the direction of the relaxation (accumulation/depletion); and (3) the upper bound of the sink reaction.

An example MATLAB workflow is also provided for the RBC data in order to demonstrate how to use the algorithm. The RBC data and model used for all analyses in this study are loaded into

MATLAB. As explained in the Methods section, PCA of the Z-scores of the raw data are taken and plotted, reproducing **Supplemental Fig. 2**. Linear regression is then performed on the data over the time intervals of the three metabolic states determined by PCA. Next, the data is input into the uFBA COBRA model building algorithm, producing a model for each state. Finally, candidate flux states through the network are determined through MCMC sampling, and the average flux through malate dehydrogenase (MDH) and ATP citrate lyase (ACITL) in **Fig. 3** are calculated.

Supplementary Figures

Supplementary Figure 1

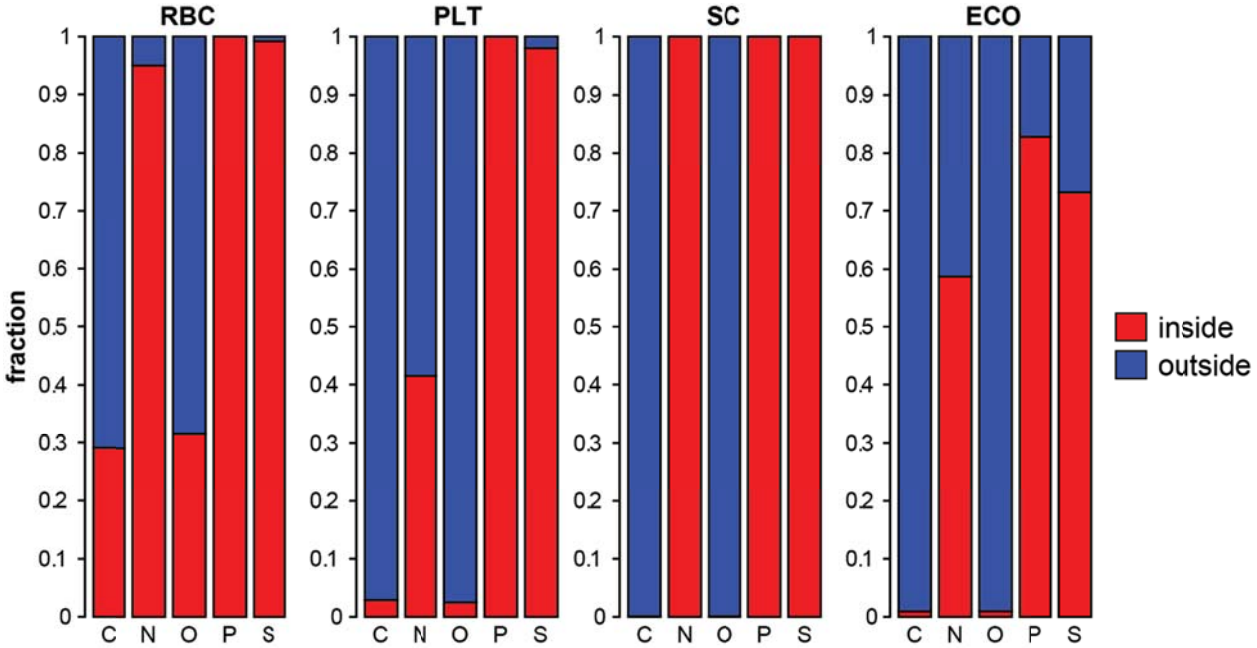

Supplementary Figure 1: Percentage of atoms in measured small metabolites across intracellular and extracellular compartments for each test system. Abbreviations: RBC, red blood cell; PLT, platelet; SC, *S. cerevisiae;* ECO, *E. coli*.

Supplementary Figure 2

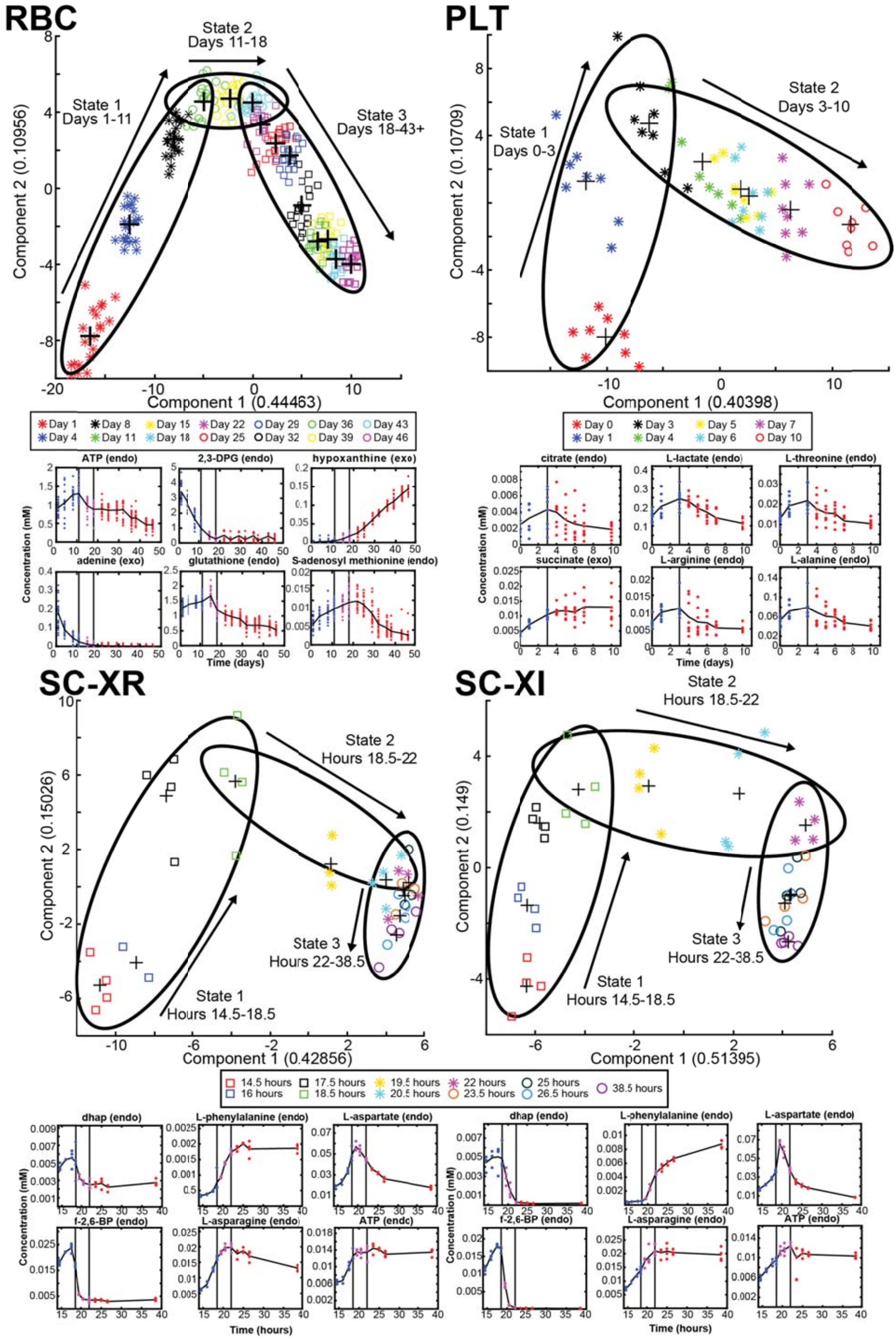

Supplementary Figure 2: Principal component analysis discretizes time-course metabolite profiles into linearized metabolic states for piecewise analysis of dynamics. PCA identifies three states for RBC, two states for platelet, and three states for each strain of *S. cerevisiae*. Some examples of small metabolite profiles are shown that best exemplify changing behavior across metabolic states. The *E. coli* data is not shown because only one state was used. Abbreviations: RBC, red blood cell; PLT, platelet; SC, *S. cerevisiae*.

Supplementary Figure 3

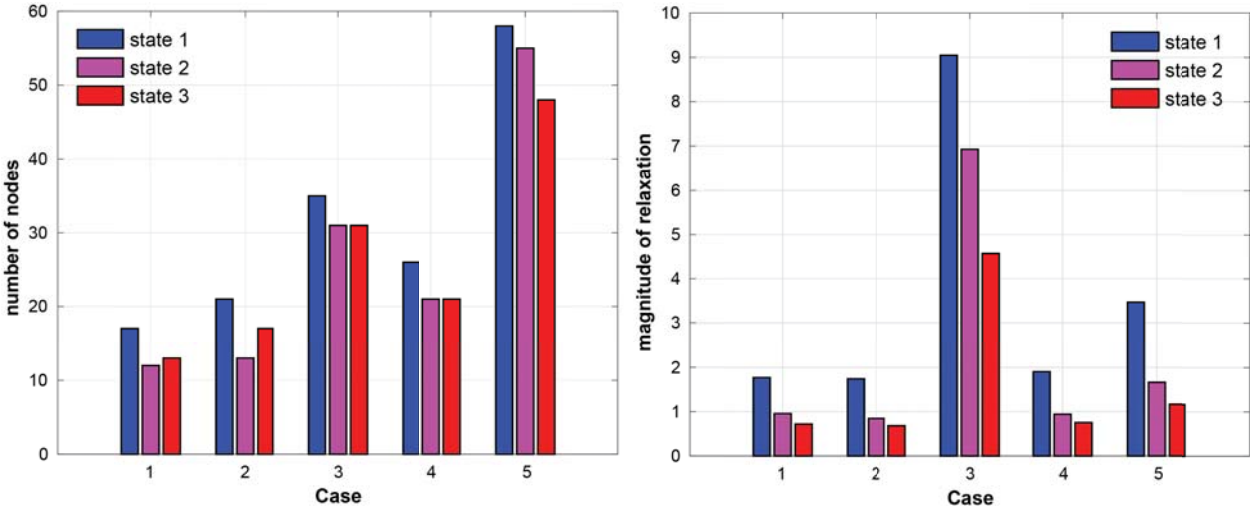

Supplementary Figure 3: Number of nodes deviating from steady-state for RBC in all three states by each of the optimization approaches (Cases). In addition, the magnitude of relaxation for the nodes is also shown. The magnitude of relaxation is defined as the sum of the absolute value of the rates of change for the metabolites deviating from steady-state.

Supplementary Figure 4

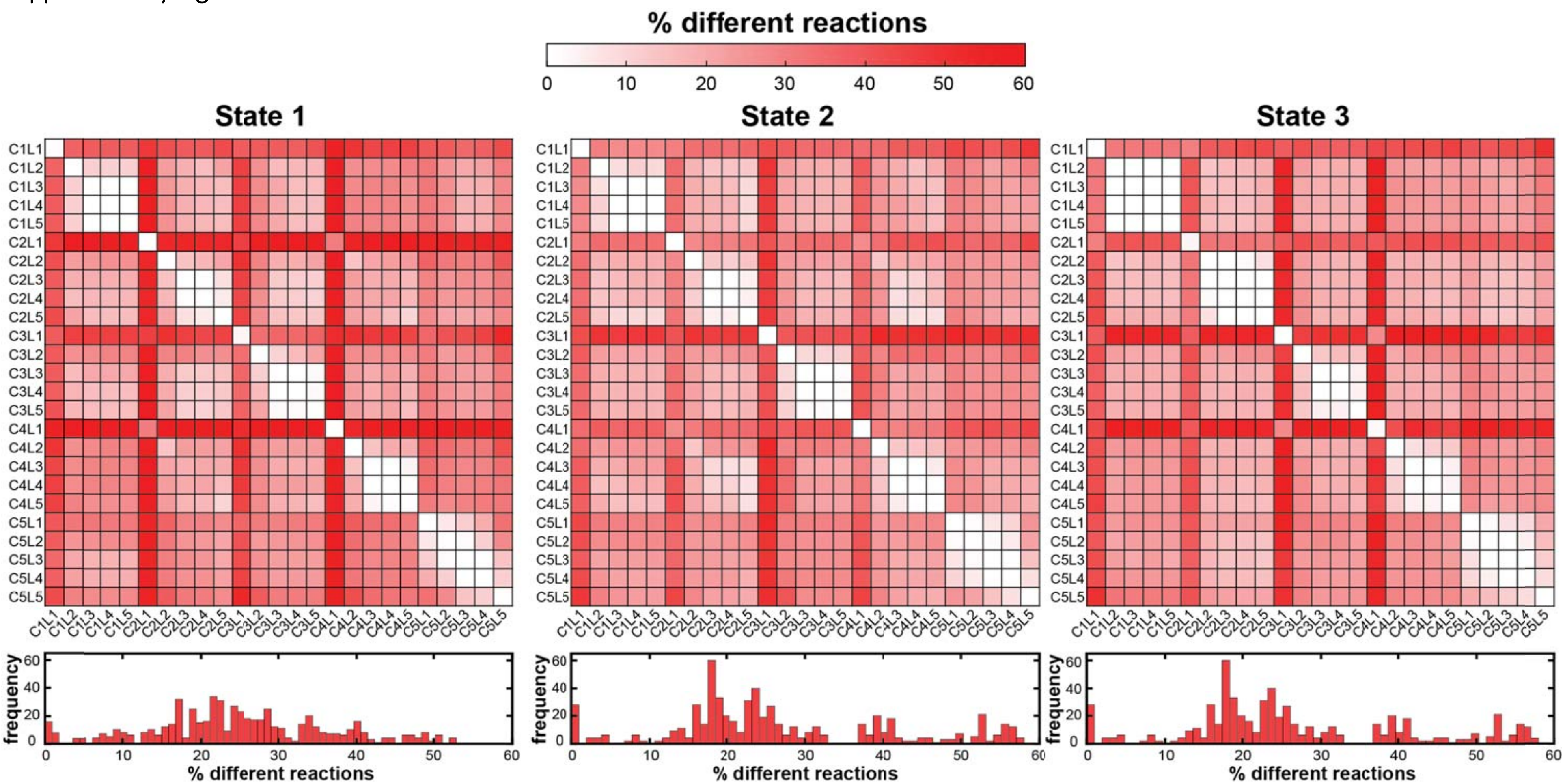

Supplementary Figure 4: Sensitivity analysis for the two parameters of metabolite relaxation algorithm for uFBA models of RBC states 1-3. The percentage of significantly different reactions based on MCMC sampling flux states is shown between models derived using the different parameters. The two parameters altered were: *case*, optimization approach; and *lambda*, magnitude of relaxation. Histograms show the distribution of percentage differences across all pairwise comparisons. Abbreviations: C1, Case 1; C2, Case 2; C3, Case 3; C4, Case 4; C5, Case 5; L1, lambda = 1; L2, lambda = 1.5; L3, lambda = 5; L4, lambda = 10; L5, lambda = 100.

Supplementary Figure 5

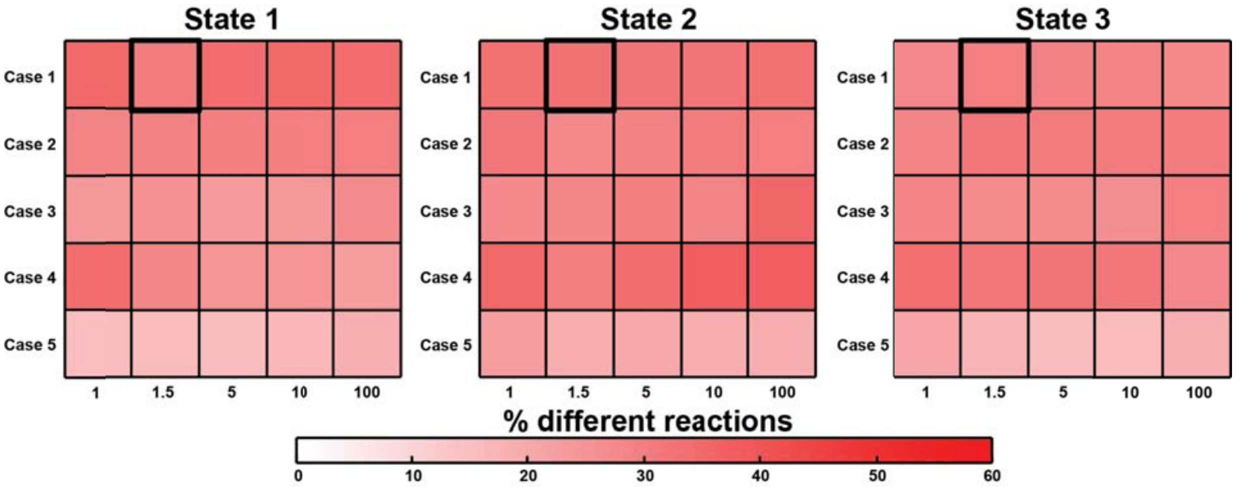

Supplementary Figure 5: Differences between uFBA and FBA flux predictions when varying parameters for metabolite node relaxation for RBC metabolic states 1-3. Optimization approaches (*cases*) are shown on the y axes and *lambda* values are shown on the x axes.

Supplementary Figure 6

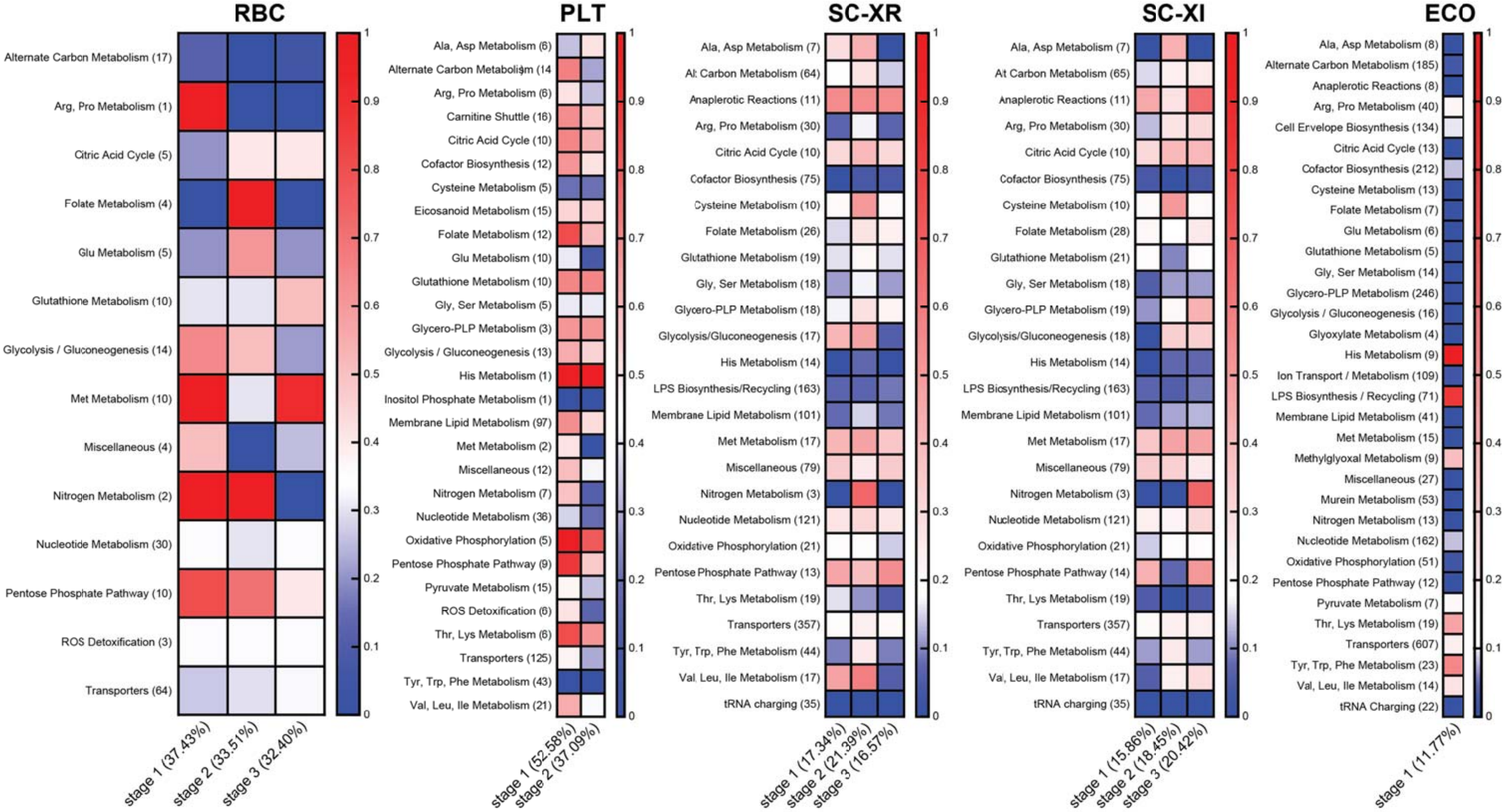

Supplementary Figure 6: Percentage of different reactions by model subsystem. The heatmap range shows the percent of different reactions between the uFBA and FBA models for the given organism and metabolic state, with white representing the global mean of different reactions across all states. Abbreviations: RBC, red blood cell; PLT, platelet; SC, *S. cerevisiae*; ECO, *E. coli*.

Supplementary Figure 7

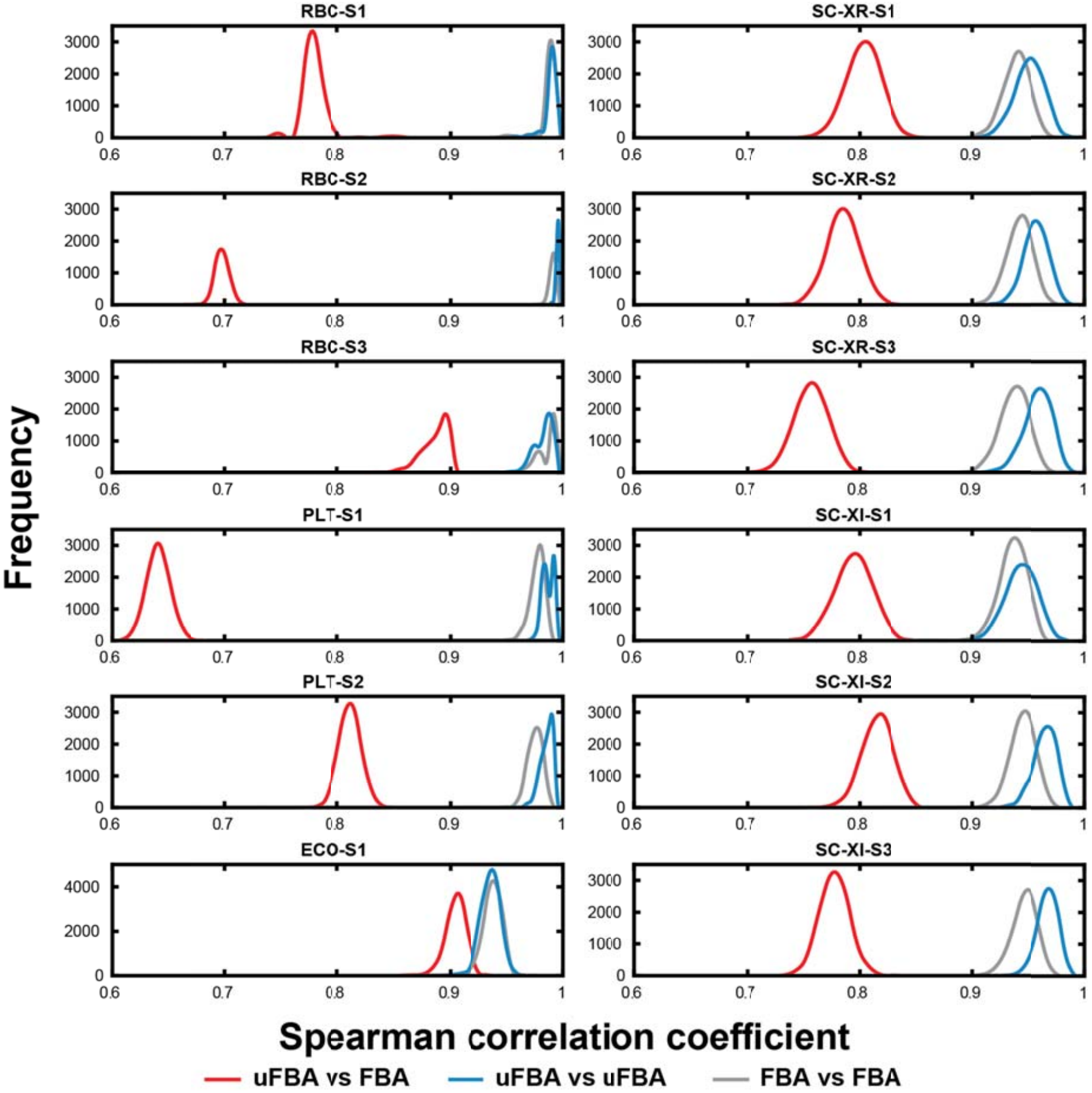

Supplementary Figure 7: Histograms of the Spearman correlation for each test case and metabolic state. The Spearman correlation between uFBA and FBA sampled fluxes (red) is lower than the Spearman correlation within a random shuffling of the reactions of both models separately. Abbreviations: RBC, red blood cell; PLT, platelet; SC, *S. cerevisiae*; ECO, *E. coli*; S1, metabolic state 1; S2, metabolic state 2; S3, metabolic state 3.

Supplementary Figure 8

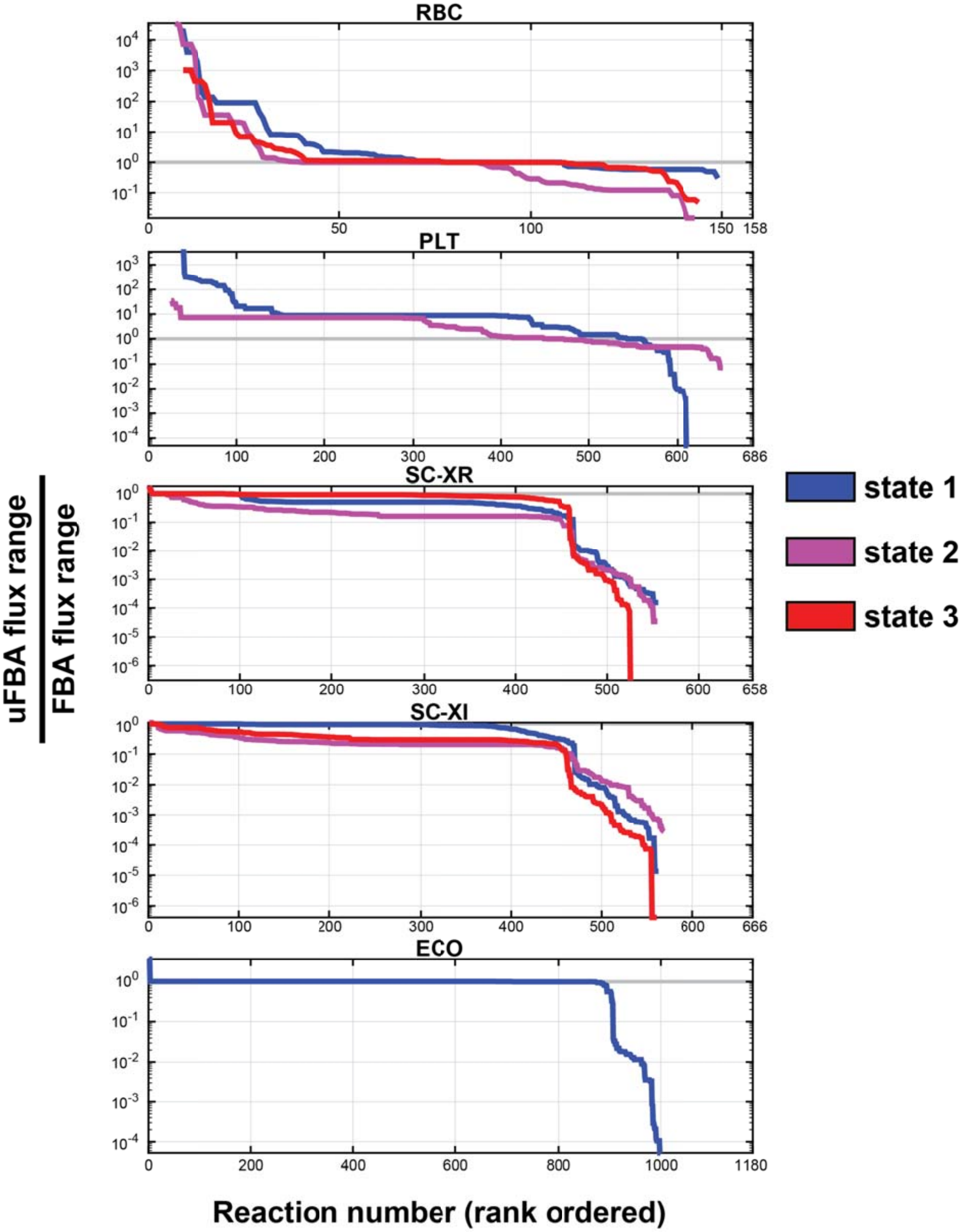

Supplementary Figure 8: Comparison of solution spaces for each system and metabolic state based on the ratio of flux ranges calculated by flux variability analysis. Grey lines denote an equal size flux range. Reactions with an infinite or zero flux range ratio are included, but no value is shown. White space denotes how many of those reactions occur. (i.e., there was allowable flux in one model but not in the other). Reactions that were unable to carry flux in both models are not shown. Abbreviations: RBC, red blood cell; PLT, platelet; SC, *S. cerevisiae*; ECO, *E. coli*.

Supplementary Figure 9

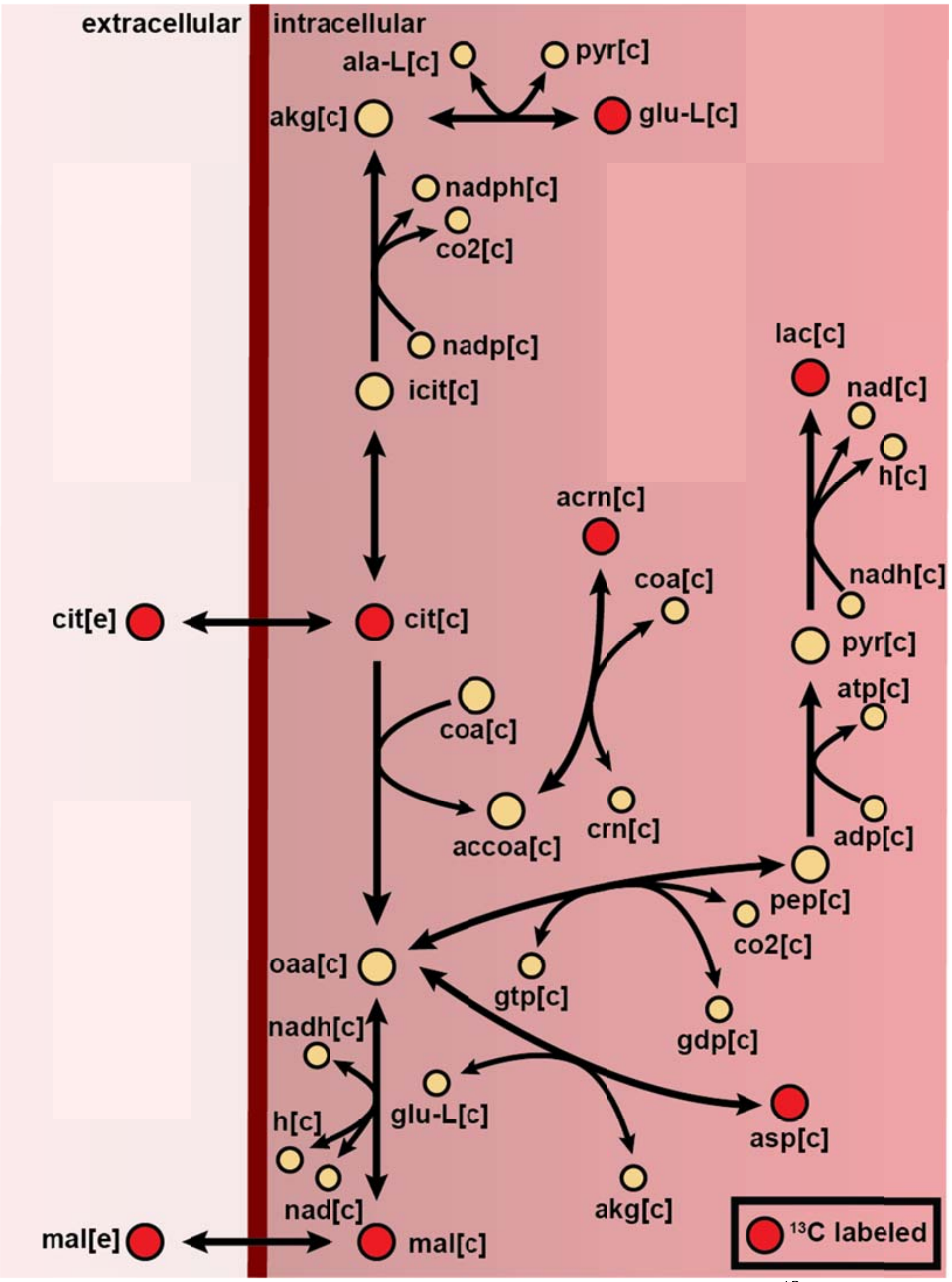

Supplementary Figure 9: Pathway map for RBC citrate metabolism and the ^{13}C labeled metabolites in RBCs after introduction of ^{13}C labeled citrate (m+6).

Supplementary Figure 10

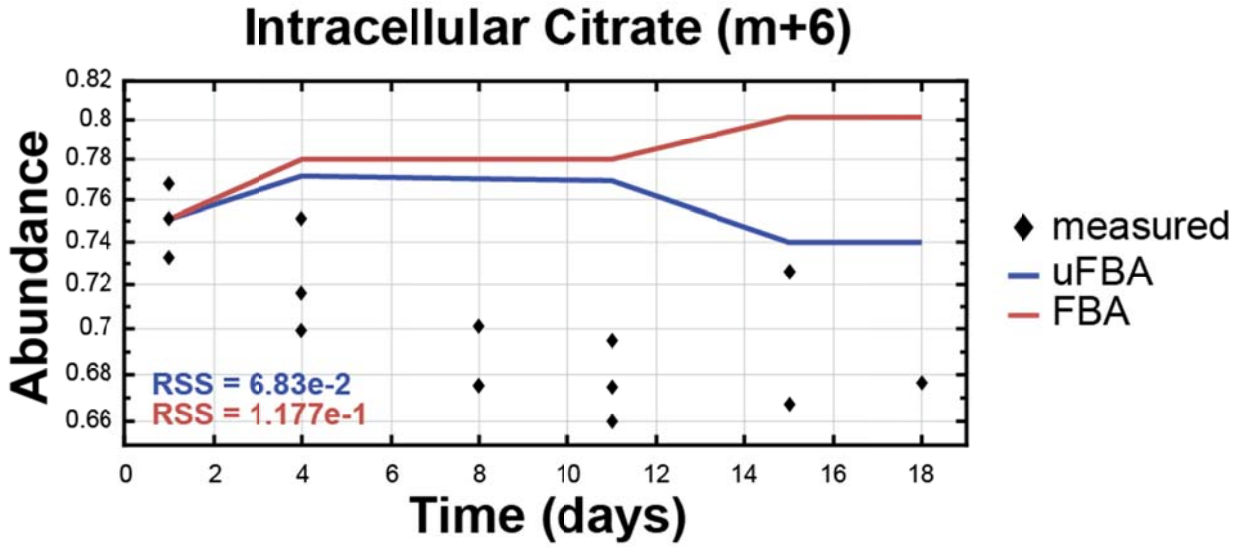

Supplementary Figure 10: MFA simulation and labeling for intracellular citrate. The residual sum of squares (RSS) is shown for both the uFBA and FBA model predicted labeling patterns.

Supplementary Figure 11

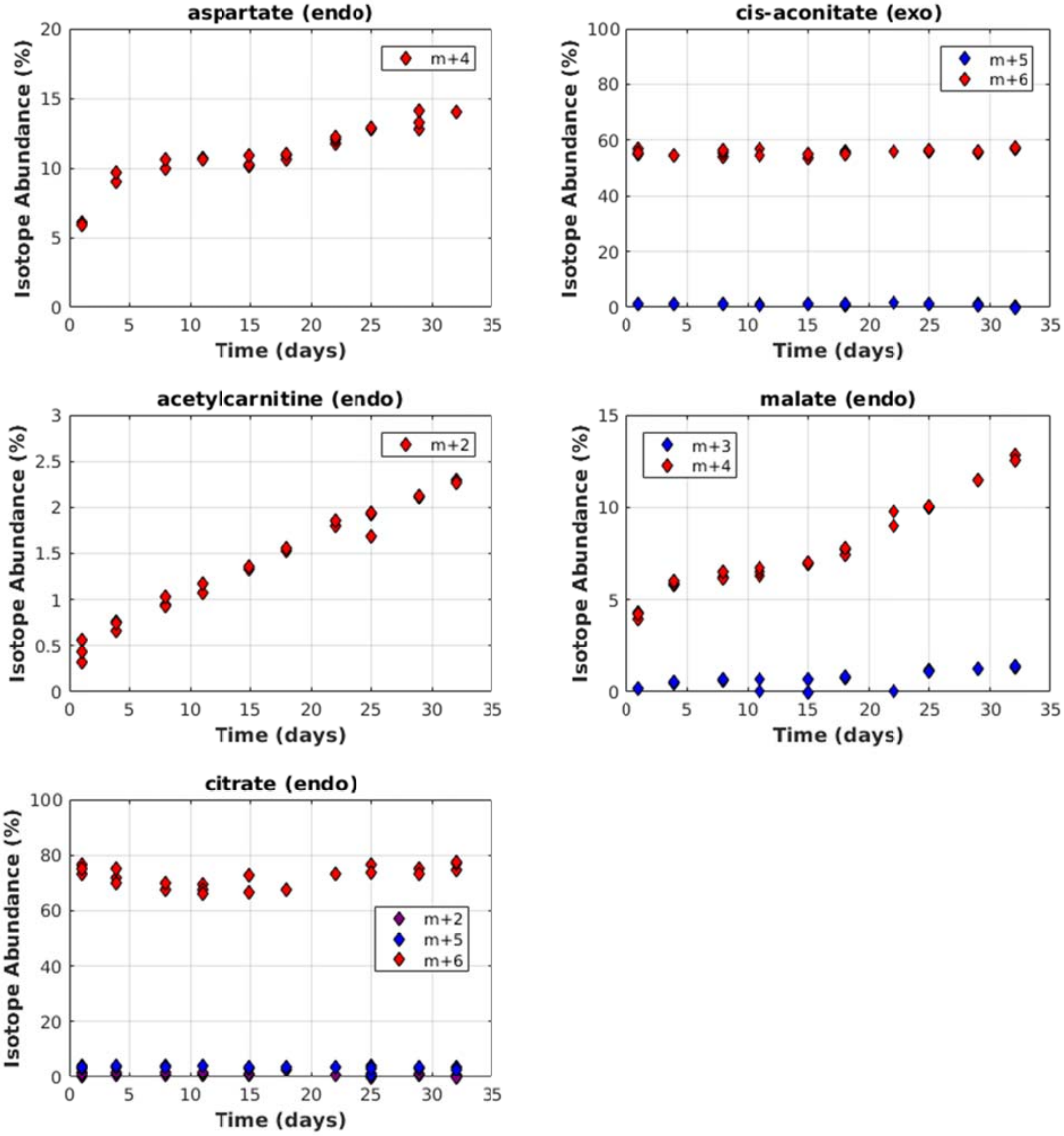

Supplementary Figure 11: Isotopic labeling of RBC metabolites that were not absolutely quantified in the original study (aspartate, cisc-aconitate, or actylcarnitine) or metabolites that had multiple detected labeling patterns (malate and citrate). Unlabeled percentages are equal to ~100 minus the sum of the total labeled amounts shown here for the specific labeling patterns.

Supplementary Figure 12

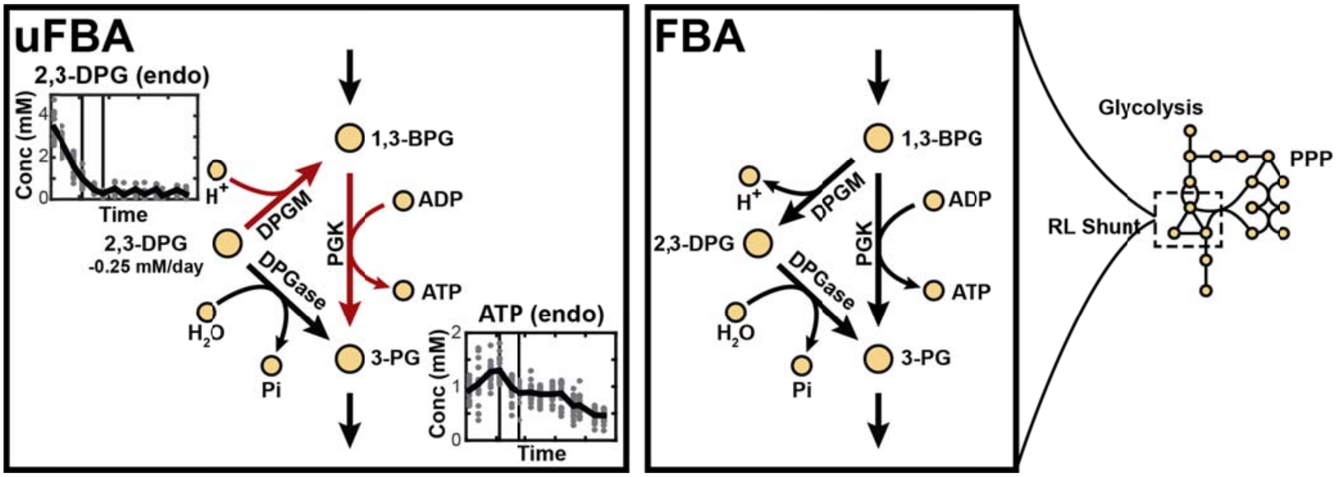

Supplementary Figure 12: The large intracellular 2,3-DPG and ATP concentrations affect flux through the Rapoport-Luebering (RL) shunt in glycolysis of RBCs. 2,3-DPG is depleting at a high rate (0.25 mM/day) in the first state, which uFBA predicts will proceed backward through the DPGM reaction to 1,3-BPG where it continues through PGK. This results in additional production of ATP, which corresponds with the spike in intracellular ATP observed at the end of the first state (day 10). The FBA model does not account for either the increasing ATP or the depleting 2,3-DPG and thus predicts flow through the RL shunt in the forward direction, toward 3-PG. Time spans 0-45 days for the inset concentration plots. Abbreviations: DPGM, diphosphoglyceromutase; PGK, phosphoglyerate kinase; DPGase, diphosphoglycerate kinase; 1,3-BPG, 1,3-bisphosphoglycerate; 2,3-DPG, 2,3-diphosphoglycerate; 3-PG, 3-phosphoglycerate.

Supplementary Tables

Supplementary Table 1: Characteristics of metabolomics data for each test system.

Cell type	# of time points measured	# of replicates	Average cell volume	Ratio of total volume that are cells (%)	# of metabolites in model	% of model metabolites measured	% of metabolites at steady-state	% of metabolites that deviate from steady-state
**Human RBC**	14	20	90 fL	60%	216	43.1%	2.78%	40.3%
**Human platelet**	8	8	10 fL	0.50%	738	14.6%	0.81%	13.8%
***S. cerevisiae* – XR Strain**	11	4	50 fL	0.25%	1228	3.91%	0.65%	3.26%
***S. cerevisiae* – XI Strain**	11	4	50 fL	0.25%	1228	3.91%	0.65%	3.26%
E. coli	3	3	1 fL	0.02%	1805	5.87%	3.27%	2.60%

Supplementary Table 2: List of model input parameters, see uFBA method documentation. Abbreviations: BiGG IDs for metabolite names; RBC, red blood cell; PLT, platelet; SC, *S. cerevisiae*; ECO, *E. coli*.

Cell type	metNoSink	metNoSinkDown	metNoSinkUp
**RBC**	abt[c], arab-L[c], arab-L[e], cmp[c], ctp[c], cdp[c], gal1p[c], gal[c], gal[e]l galt[c], gam6p[c], gam[c], gam[3], gluala[e], nac[c], nac[e], orot5p[c], orot[c], orot[e], rib-D[c], rib-D[e], udp[c], utp[c], ump[c], xylt[c], xylt[e], xylu-D[c], xylu-D[e], xylu-L[c]	adn[e], akg[e], gsn[e]	N/A
**PLT**	abt[c], acac[m], arab_L[c], arab_L[e], cmp[c], ctp[c], cdp[c], fald[m], gal1p[c], gal[c], gal[e], galt[c], gam6p[c], gam[c], gam[e], gluala[e], gmp[e], h2o2[e], nac[c], nac[e], o2s[e], orot5[c], orot[c], orot[e], pram[c], rib_D[c], rib_D[e], xylt[c], xylt[e], xylu_D[c], xylu_D[e], xylu_L[c]	ade[e]	N/A
**SC-XR**	o2[c], o2[e], o2[m], o2[r], o2[x]	N/A	N/A
**SC-XI**	o2[c], o2[e], o2[m], o2[r], o2[x]	N/A	N/A
**ECO**	N/A	no2[e], no3[e]	N/A

Supplementary Table 3: Statistics for comparison of metabolite node relaxation algorithm with qualitative validation data. TP/FN/FP/TN and TPR values are given for when a positive prediction refers to a metabolite remaining at steady-state. Overall accuracy is given for the average of accuracy of all three categories of positive predictions.

Optimization technique	State	True positives (TP)	False negatives (FN)	False positives (FP)	True negatives (TN)	True positive rate (TPR)	Overall accuracy
**Case 1**	1<br>2	21<br>24	0<br>2	15<br>12	3<br>1	1.00<br>0.923	0.680<br>0.709
**Case 2**	1<br>2	20<br>23	1<br>3	14<br>12	4<br>1	0.952<br>0.885	0.677<br>0.686
**Case 3**	1<br>2	16<br>17	5<br>9	7<br>9	11<br>4	0.762<br>0.654	0.691<br>0.588
**Case 4**	1<br>2	11<br>23	10<br>3	7<br>11	11<br>2	0.524<br>0.885	0.565<br>0.706
**Case 5**	1<br>2	12<br>18	9<br>8	6<br>5	12<br>8	0.571<br>0.692	0.579<br>0.644

Supplementary Table 4: FBA objectives for each test system. Each model was optimized to calculate the maximum flux through the given objective reaction.

RBC		
**State 1**		
**Objective**	**uFBA**	**FBA**
NaKt	0.594	1.023
DM_nadh	0.125	0.419
GTHP	0.390	0.000
**State 2**		
**Objective**	**uFBA**	**FBA**
NaKt	0.111	0.914
DM_nadh	0.022	0.616
GTHP	0.762	0.000
**State 3**		
**Objective**	**uFBA**	**FBA**
NaKt	0.512	0.452
DM_nadh	0.068	0.331
GTHP	0.078	0.000

PLT		
**State 1**		
**Objective**	**uFBA**	**FBA**
ATPS4m	10.687	0.042
DM_nadh	0.377	10.228
**State 2**		
**Objective**	**uFBA**	**FBA**
ATPS4m	7.604	2.257
DM_nadh	0.165	3.614

SC-XR		
**State 1**		
**Objective**	**uFBA**	**FBA**
Biomass	0.269	0.431
ATPM	12.621	24.723
DM_nadh	135.342	46.948
DM_xu5p	10.312	10.547
**State 2**		
**Objective**	**uFBA**	**FBA**
Biomass	0.054	0.190
ATPM	1.851	11.361
DM_nadh	45.218	18.540
DM_xu5p	1.851	4.545
**State 3**		
**Objective**	**uFBA**	**FBA**
Biomass	0.081	0.085
ATPM	5.102	5.562
DM_nadh	22.476	5.283
DM_xu5p	2.084	1.830

SC-XI		
**State 1**		
**Objective**	**uFBA**	**FBA**
Biomass	0.311	0.308
ATPM	15.366	16.228
DM_nadh	87.103	27.277
DM_xu5p	6.263	6.120
**State 2**		
**Objective**	**uFBA**	**FBA**
Biomass	0.033	0.055
ATPM	0.430	2.078
DM_nadh	10.826	6.270
DM_xu5p	0.235	0.779
**State 3**		
**Objective**	**uFBA**	**FBA**
Biomass	0.023	0.071
ATPM	1.478	4.932
DM_nadh	19.011	8.160
DM_xu5p	1.691	2.228

ECO		
**State 1**		
**Objective**	**uFBA**	**FBA**
Biomass	0.857	0.850
ATPM	105.878	104.173
DM_nadh	45.657	44.899

Supplementary Table 5: List of ^{13}C labeled metabolites.

Metabolite	Location	# Carbons labeled (# Carbons total)
Acetylcarnitine	Intracellular	2 (9)
Aspartate	Intracellular	4 (4)
Citrate	Intracellular, extracellular	6 (6)
Glutamate	Intracellular	5 (5)
Lactate	Intracellular	3 (3)
Malate	Intracellular, extracellular	4 (4)

Supplementary Table 6: Flux through RL shunt reactions with additional MIPP1 reaction.

Reaction	Reaction stoichiometry	Flux (mM/day)
**DPGM**	2,3-DPG + H → 1,3-BPG	0.2040
**DPGase**	2,3-DPG + H_2O → 3-PG + phosphate	0.0118
**MIPP1**	2,3-DPG + H_2O → 2-PG + phosphate	0.0125

Supplementary Table 7: Predicted and experimental oxygen uptake rate, electron transport chain usage, and ATP production in the platelet.

Method	State	Oxygen uptake rate (mM/day)	ETC ATP generation (mM/day)	% ATP production from ETC
FBA	1<br>2	0.0154<br>0.616	0.0105<br>2.07	0.23%<br>64.1%
uFBA	1<br>2	2.98<br>2.16	9.80<br>7.07	90.2%<br>88.8%
Experimental[13,14]	1<br>2	2.34 +/- 0.403<br>2.14 +/- 0.340	11.4	87.5%<br>86.1%

Supplementary Table 8: Comparison of glycolysis and oxidative pentose phosphate pathway fluxes in *S. cerevisiae*.

	Method	State	Hexokinase flux (mmol/gDW/h)	G6PDH flux (mmol/gDW/h)	% of glucose shuttled to PPP
**XI strain**	**uFBA**	1<br>2<br>3	6.3067<br>0.97273<br>0.032322	1.0069<br>0.058757<br>0.53959	15.966%<br>6.0404%<br>-
	**FBA**	1<br>2<br>3	6.1542<br>0.96022<br>0.080397	0.40367<br>0.051064<br>0.17895	6.5486%<br>5.3179%<br>-
**XR strain**	**uFBA**	1<br>2<br>3	9.2686<br>0.59132<br>0.24106	1.0133<br>0.27492<br>0.85177	10.932%<br>46.492%<br>-
	**FBA**	1<br>2<br>3	12.854<br>2.8191<br>0.19376	0.11955<br>0.070963<br>0.067916	0.93004%<br>2.5172%<br>35.052%

Supplementary Table 9: Comparison of predicted flux state to measured flux state for *S. cerevisiae* strain XR under glucose growth (State 1). Flux values are normalized to 100 units for glucose uptake (HEX1).

Reaction	Measured[8]	Measured STD[8]	uFBA	FBA
HEX1	100.0000	0.0000	0.0349	0.0296
PGI	73.1818	16.1000	178.1791	121.2232
PFK	81.5909	4.8955	64.3092	67.8593
FBA	81.5909	4.8955	63.2282	66.6181
TPI	81.5909	4.8955	95.4335	109.9102
PGK	167.0455	5.0114	242.5295	263.2137
PGM	161.5909	4.8477	241.0144	260.7549
PYK	185.2273	1.8523	238.4090	257.9616
G6PDH2	19.7727	4.5477	10.9320	0.9300
GND	19.7727	4.5477	10.9355	0.9300
RPE	8.4091	1.9341	12.8204	23.6504
RPI	11.5909	2.5500	23.7492	24.5804
TKT1	4.3182	0.9932	23.2652	21.8701
TKT2	3.8636	0.8886	21.4153	19.9001
TALA	4.3182	0.9932	39.0313	44.6174
PC	34.3182	6.1773	33.8459	51.0278
PPCK	24.0909	6.2636	1.0946	1.1467
PYRt2m	10.2273	3.0682	60.5392	409.9270
PDHm	15.9091	3.8182	7.8786	3.9529
CSm	46.3636	3.2455	1.8255	3.2267
ICDHxm	46.3636	3.2455	1.8255	3.2267
AKGDbm	45.4545	3.6364	0.4031	0.5733
SUCOASm	45.4545	3.6364	0.0036	2.8521
SUCD1m	45.4545	3.6364	0.0036	2.8521
PYRDC	128.4091	3.8523	12.3039	12.2219
ALDD2y	18.1818	3.6364	204.8390	186.2334

Supplementary Table 10: Comparison of predicted flux state to measured flux state for *S. cerevisiae* strain XR under xylose growth (State 3). Flux values are normalized to 100 units for xylose uptake (XYLR).

Reaction	Measured[8]	Measured STD[8]	uFBA	FBA
HEX1	0.0000	0.0000	0.0349	0.0296
XYLR	100.0000	0.0000	100.0000	100.0000
PGI	53.8462	14.5385	0.0218	4.9936
PFK	46.1538	4.1538	0.1377	0.1557
FBA	46.1538	4.1538	0.1321	0.1508
TPI	46.1538	4.1538	0.2697	0.3017
PGK	146.1538	5.8462	0.6250	0.6513
PGM	138.4615	5.5385	0.6151	0.6451
PYK	169.2308	52.4615	0.6044	0.6199
G6PDH2	53.8462	14.5385	0.1259	0.0095
GND	53.8462	14.5385	0.1259	0.0095
RPE	0.0000	0.0000	0.0310	0.0643
RPI	53.8462	4.8462	0.0948	0.0738
TKT1	53.8462	4.8462	0.0930	0.0550
TKT2	53.8462	4.8462	0.0849	0.0400
TALA	53.8462	4.8462	0.0446	0.1017
PC	138.4615	36.0000	0.1412	0.1004
PPCK	30.7692	1.2308	0.0056	0.0047
PYRt2m	0.0000	0.0000	1.0688	1.6527
OAAt2m	107.6923	4.3077	99.7038	102.5414
PDHm	107.6923	4.3077	0.0408	0.0245
CSm	130.7692	5.2308	0.0122	0.0220
ICDHxm	130.7692	5.2308	0.0028	0.0050
AKGDbm	130.7692	5.2308	0.0000	0.0450
SUCOASm	130.7692	5.2308	0.0000	0.0450
SUCD1m	130.7692	5.2308	75.8868	73.7246
FUMm	130.7692	5.2308	45.5365	47.1617
MDHm	23.0769	6.6923	49.9257	49.1885
ME2m	107.6923	4.3077	0.0570	0.0347
PYRDC	23.0769	6.9231	0.3409	0.3105
ALDD2y	23.0769	6.9231	0.0063	0.0286

Supplementary Table 11: Comparison of predicted flux state to measured flux state for *S. cerevisiae* strain XI under glucose growth (State 1). Flux values are normalized to 100 units for glucose uptake (HEX1).

Reaction	Measured[7]	Measured 95% LB[7]	Measured 95% UB[7]	uFBA	FBA
HEX1	100.0000	89.1698	111.2083	100.0000	100.0000
XI	0.0000	0.0000	6.8028	0.0000	0.0000
PGI	92.0682	81.8755	102.4833	36.8263	206.9947
PFK	93.5508	82.9281	104.4626	91.3782	93.6667
FBA	93.5508	82.9281	104.4626	89.5159	92.1374
TPI	71.3862	62.1890	82.0163	158.9041	163.8829
PGK	166.1972	146.4196	187.5760	343.1248	349.8168
PGM	165.6783	146.0044	187.0052	341.7403	348.2240
PYK	164.7887	145.1149	186.1082	339.4876	345.8851
G6PDH2	5.0408	4.2506	6.2691	15.5930	6.4353
G3PD1ir	22.1646	17.9600	25.8428	28.2988	31.8310
G3PT	22.0904	17.9274	25.7999	30.1328	28.7418
PYRDC	157.4500	137.5315	179.8221	325.5402	292.8302
THRAi	1.0378	0.0000	1.5901	0.9950	0.9323
RPI	2.2239	1.9325	2.6064	6.3776	5.0035
TKT1	4.4477	3.6998	5.7183	5.6369	1.6592
TKT2	4.0030	3.9251	4.5975	3.5765	0.2276
TALA	3.2617	2.7650	4.1097	85.2436	92.0471
PC	5.7079	3.7265	8.1394	33.4511	50.2061
PYRt2m	1.4826	0.0000	5.5182	10.3818	119.6122
CSm	1.7050	1.6783	1.7761	1.6730	2.9576
ICDHxm	1.7050	1.6783	1.7761	0.4011	0.4620
SUCOASm	0.0000	0.0000	0.0193	0.0029	3.4103
ME2m	1.3343	0.0815	6.0089	7.8215	7.9244

Supplementary Table 12: Normalized errors for predicted flux state to measured flux state for *S. cerevisiae*.

Model	XR		XI
	Glucose (State 1)	Xylose (State 3)	Glucose (State 1)
**uFBA**	0.4799	0.9090	0.9675
**FBA**	0.5616	0.9100	1.0484

Supplementary Table 13: Characteristics of conflicting gene essentiality in *E. coli*.

**Metabolic Pathway**	**Gene**	**Essential (uFBA)**	**Essential (FBA)**	**LB Essential**	**Orth et al. 1**	**Orth et al. 2**	**Baba et al.**	**Patrick et al.**
**NAD biosynthesis**	b0109	N	Y		Y	Y	N	Y
	b0639	N	Y	Y				
	b0750	N	Y		Y	Y	N	Y
	b1740	N	Y	Y				
	b2574	N	Y		Y	Y	N	Y
	b2615	N	Y	Y				
**Purine biosynthesis**	b0522	N	Y		Y	Y	N	Y
	b0523	N	Y		Y	Y	Y	Y
	b1131	N	Y	Y				
	b2476	N	Y		N	N	Y	Y
	b4177	N	Y		N	N	Y	Y

References

1 Bergdahl, B., Heer, D., Sauer, U., Hahn-Hagerdal, B. & van Niel, E. W. Dynamic metabolomics differentiates between carbon and energy starvation in recombinant Saccharomyces cerevisiae fermenting xylose. *Biotechnol Biofuels* **5**, 34, doi:10.1186/1754-6834-5-34 (2012).

2 Mahadevan, R. & Schilling, C. H. The effects of alternate optimal solutions in constraint-based genome-scale metabolic models. *Metab Eng* **5**, 264-276 (2003).

3 Bordbar, A. *et al.* Identified metabolic signature for assessing red blood cell unit quality is associated with endothelial damage markers and clinical outcomes. *Transfusion* **56**, 852-862, doi:10.1111/trf.13460 (2016).

4 Veale, M. F. *et al.* AS-7 improved in vitro quality of red blood cells prepared from whole blood held overnight at room temperature. *Transfusion* **55**, 108-114, doi:10.1111/trf.12779 (2015).

5 Cho, J., King, J. S., Qian, X., Harwood, A. J. & Shears, S. B. Dephosphorylation of 2,3-bisphosphoglycerate by MIPP expands the regulatory capacity of the Rapoport-Luebering glycolytic shunt. *Proc Natl Acad Sci U S A* **105**, 5998-6003, doi:10.1073/pnas.0710980105 (2008).

6 Frick, O. & Wittmann, C. Characterization of the metabolic shift between oxidative and fermentative growth in Saccharomyces cerevisiae by comparative 13C flux analysis. *Microb Cell Fact* **4**, 30, doi:10.1186/1475-2859-4-30 (2005).

7 Wasylenko, T. M. & Stephanopoulos, G. Metabolomic and (13)C-metabolic flux analysis of a xylose-consuming Saccharomyces cerevisiae strain expressing xylose isomerase. *Biotechnol Bioeng* **112**, 470-483, doi:10.1002/bit.25447 (2015).

8 Feng, X. Y. & Zhao, H. M. Investigating glucose and xylose metabolism in Saccharomyces cerevisiae and Scheffersomyces stipitis via C-13 metabolic flux analysis. *Aiche J* **59**, 3195-3202, doi:10.1002/aic.14182 (2013).

9 Orth, J. D. *et al.* A comprehensive genome-scale reconstruction of Escherichia coli metabolism--2011. *Mol Syst Biol* **7**, 535, doi:10.1038/msb.2011.65 (2011).

10 Baba, T. *et al.* Construction of *Escherichia coli* K-12 in-frame, single-gene knockout mutants: the Keio collection. *Mol Syst Biol* **2**, 2006.0008 (2006).

11 Patrick, W. M., Quandt, E. M., Swartzlander, D. B. & Matsumura, I. Multicopy suppression underpins metabolic evolvability. *Mol Biol Evol* **24**, 2716-2722, doi:10.1093/molbev/msm204 (2007).

12 Schellenberger, J. *et al.* Quantitative prediction of cellular metabolism with constraint-based models: the COBRA Toolbox v2.0. *Nat Protoc* **6**, 1290-1307, doi:10.1038/nprot.2011.308 (2011).

13 Picker, S. M., Schneider, V., Oustianskaia, L. & Gathof, B. S. Cell viability during platelet storage in correlation to cellular metabolism after different pathogen reduction technologies. *Transfusion* **49**, 2311-2318, doi:10.1111/j.1537-2995.2009.02316.x (2009).

14 Kilkson, H., Holme, S. & Murphy, S. Platelet metabolism during storage of platelet concentrates at 22 degrees C. *Blood* **64**, 406-414 (1984).
